# Whole-cell energy modeling reveals quantitative changes of predicted energy flows in RAS mutant cancer cell lines

**DOI:** 10.1016/j.isci.2023.105931

**Published:** 2023-01-05

**Authors:** Thomas Sevrin, Lisa Strasser, Camille Ternet, Philipp Junk, Miriam Caffarini, Stella Prins, Cian D’Arcy, Simona Catozzi, Giorgio Oliviero, Kieran Wynne, Christina Kiel, Philip J. Luthert

**Affiliations:** 1 Systems Biology Ireland, School of Medicine, University College Dublin, Belfield Dublin 4, Ireland; 2 UCD Charles Institute of Dermatology, School of Medicine, University College Dublin, Belfield Dublin 4, Ireland; 3 UCL Institute of Ophthalmology, University College London, 11-43 Bath Street, London EC1V 9EL, UK; 4 Conway Institute of Biomolecular & Biomedical Research, University College Dublin, Dublin 4, Ireland; 5 Department of Molecular Medicine, University of Pavia, 27100 Pavia, Italy; 6 NIHR Moorfields Biomedical Research Centre, University College London, 11-43 Bath Street, London EC1V 9EL, UK

**Keywords:** Protein, Cellular physiology

## Abstract

Cellular utilization of available energy flows to drive a multitude of forms of cellular “work” is a major biological constraint. Cells steer metabolism to address changing phenotypic states but little is known as to how bioenergetics couples to the richness of processes in a cell as a whole. Here, we outline a whole-cell energy framework that is informed by proteomic analysis and an energetics-based gene ontology. We separate analysis of metabolic supply and the capacity to generate high-energy phosphates from a representation of demand that is built on the relative abundance of ATPases and GTPases that deliver cellular work. We employed mouse embryonic fibroblast cell lines that express wild-type KRAS or oncogenic mutations and with distinct phenotypes. We observe shifts between energy-requiring processes. Calibrating against Seahorse analysis, we have created a whole-cell energy budget with apparent predictive power, for instance in relation to protein synthesis.

## Highlights

• A whole-cell energetics model is described for MEF cells with different Ras mutations
• Shifts in metabolism reflect both changes in protein abundances and energy supply
• A whole-cell energy budget is created with predictive capacity

## Graphical abstract

**Figure:**
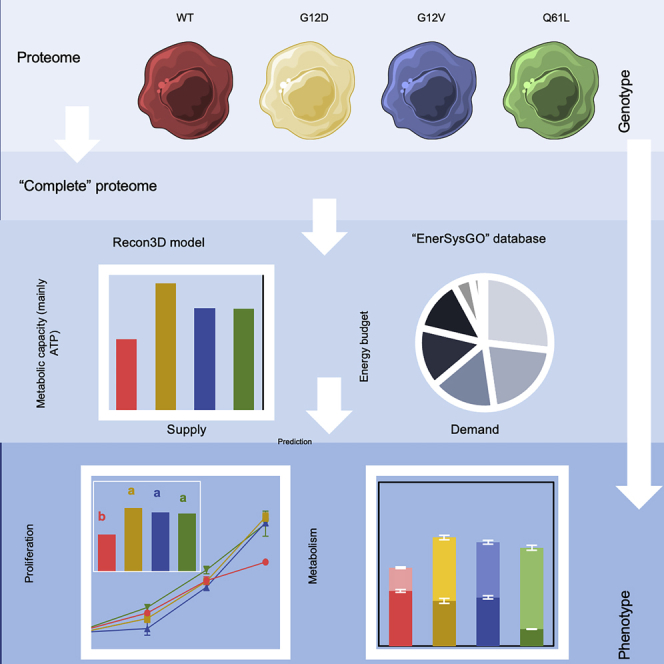

## Introduction

Cells are highly constrained in both time and space by the laws of physics, including those of thermodynamics. For example, there is a limit to the packing density of transporters in a membrane, a limit to the rate of any biochemical reaction, and a limit to the rate of delivery of a metabolite from outside the cell by diffusion. A critical constraint is the maximum capacity for cell (and organism) work using chemical energy (bioenergetics). This constraint and the forces of natural selection have led most biological systems to be highly optimized,^1^ particularly with regard to bioenergetics as, in general, high-energy sources and (for aerobic species) oxygen supply are limiting factors. Recently, it has been suggested that dissipation of Gibbs free energy is a further constraint.^2^ Cells deliver multiple, potentially competing, processes and need to distribute available energy between those processes in an optimized way.^3^ Therefore, we argue that it is essential to understand the cell as a whole in order to appreciate the total energy demands that have to be met.^4^ This approach aligns with the emerging field of physical bioenergetics.^3^

Proteomics can be used to capture whole-cell properties and protein-protein interaction networks and indeed other network descriptors can provide further insights but, in general, these approaches do not accommodate the intrinsic constraints imposed by considerations of bioenergetics. We have established a framework with which to consider energy flows that includes separate treatments of energy supply (high-energy phosphates) and demand (ATPases) and is informed by proteomic data. To model energy supply, we elected to use a genome-scale metabolic model (GEM), together with constraint-based reconstruction and analysis (COBRA) methods.^5^ These tools have been proven powerful over the last decade to predict metabolic fluxes, and they are appealing approaches for probing physiology and disease mechanisms.^6^^,^^7^^,^^8^ A strength of these methods is that they have the capacity to embrace the entire cell, tissue, or even organism. The Recon3D model is a comprehensive human metabolic network and contains 2,248 proteins and 2,797 metabolites that are linked via 10,600 reactions.^9^ A key part of the COBRA methodology is finding the steady-state solution to the product of the reaction stoichiometric matrix defined in the metabolic network and the vector of fluxes through all the reactions (including transport as a specific class of reaction that moves a metabolite from one compartment to another). The solution space is intrinsically vast and it is reduced by first constraining the lower and upper flux bounds for each reaction and then solving to optimize a so-called “objective function”, e.g. the accumulation of biomass or ATP production. Upper and lower flux bounds can be constrained based on gene expression levels (for reactions that involve enzymes or transporters) and/or nutrient availability from the growth medium (so-called “exchange reactions”).^10^

Using constraint-based methods to predict phenotype is challenging in the context of complex mammalian cells. The intrinsic need to select an objective function means that in general only a single process can be optimized whereas in a cell or tissue there is “competition” for resources between a very large number of processes that in turn drive phenotypic characteristics of the system. Cells need to maintain ion gradients, turnover proteins (production, secretion, degradation, and post-translational modifications), lipids, and carbohydrates as well as execute other energy requiring, specialized functions.^11^^,^^12^

How cells operate and which cellular processes take place is largely driven by proteins, which are the executors of the cell’s genome. It is now possible to obtain high-quality, deep-coverage, and quantitative proteomics data in a routine fashion in human cells and tissues.^13^^,^^14^ In this work, we test the hypothesis that the relative abundance of ATPases combined with modeling of capacity to generate ATP can offer a useful approach in the study of whole-cell/whole-tissue systems. We developed EnerSysGO (energy-centric systematic gene ontology), a database that classifies proteins according to their need for high-energy phosphates (ATP, GTP [guanosine triphosphate], …) or reducing equivalents (NAD+ [Nicotinamide adenine dinucleotide], NADP+ [Nicotinamide adenine dinucleotide phosphate],..) to execute cellular processes.

To explore the potential validity of this framework, we sought to find a system where metabolic variation associated with differing phenotypes could be imposed on a relatively uniform background. To that end, we have elected to use mouse embryonic fibroblast (MEF) cell lines with different mutations in rat sarcoma virus (Ras) protein. Ras oncoproteins, with the isoforms HRAS (Harvey rat sarcoma viral oncogene homolog), KRAS (Kirsten rat sarcoma virus), and NRAS (neuroblastoma Ras viral oncogene homolog), are small GTPases that cycle between active guanosine triphosphate (GTP)-bound and inactive guanosine diphosphate (GDP)-bound states.^15^ In that cycle, guanine nucleotide exchange factors (GEFs) promote activation of Ras, and GTPase-activating proteins (GAPs) inactivate Ras by catalyzing the intrinsically slow GTP hydrolysis rate. In the active state, Ras proteins interact with several effector proteins, which cause the activation of several downstream effector pathways (Figure S1A). Hence, Ras proteins are key cellular switches that control whole-cell phenotypes, such as proliferation, differentiation, survival, migration, and apoptosis. Among the three Ras isoforms, mutations in KRAS predominate in lung, colorectal, and pancreatic cancer (Figure S1B).^16^^,^^17^ Over the past years, several studies provided evidence that different types of (K)RAS mutations (e.g. G12D, G12V, and G13D) exhibit variations in their phenotypic manifestations, such as transforming potential and migration properties.^18^^,^^19^^,^^20^^,^^21^^,^^22^^,^^23^^,^^24^^,^^25^^,^^26^ Furthermore, (K)RAS mutant- and copy number-specific alterations in lipid, nucleotide, and glycolytic metabolism have been established in both *in vitro* and *in vivo* systems.^27^^,^^28^^,^^29^^,^^30^

We predicted that all KRAS mutant MEF cell lines have increased capacity to produce ATP, but there are also modest shifts between energy-requiring processes, possibly arising because the total capacity is limited. We propose that an integrated approach to mapping and estimating energy flows in cells is critical to understanding how cells function and respond to disease-related perturbations.

## Results

### A whole-cell energetics framework

To try to capture whole-cell energetics in a way that could be informed by our proteomic analysis and predict at least some processes, we established a simple whole-cell model of metabolism that is built around ATP synthesis. This consists of a supply model that predicts the maximum capacity of the cell to generate ATP and a demand side that predicts energy utilization. For the former, we used flux balance analysis (see STAR Methods). The central assumption of the demand side model is that ATP flux is distributed between all the ATP-requiring enzymes within a cell in proportion to abundance (corrected for complexes and, where possible, turnover rate kcat, see STAR Methods). Clearly, this approach fails to take into account many additional variables, including substrate availability and related thermodynamic constraints, but we sought to understand whether or not this simple approach had predictive power.

### Genotype-phenotype relation in KRAS wild type and mutant MEF cell lines

**Figure 1:**
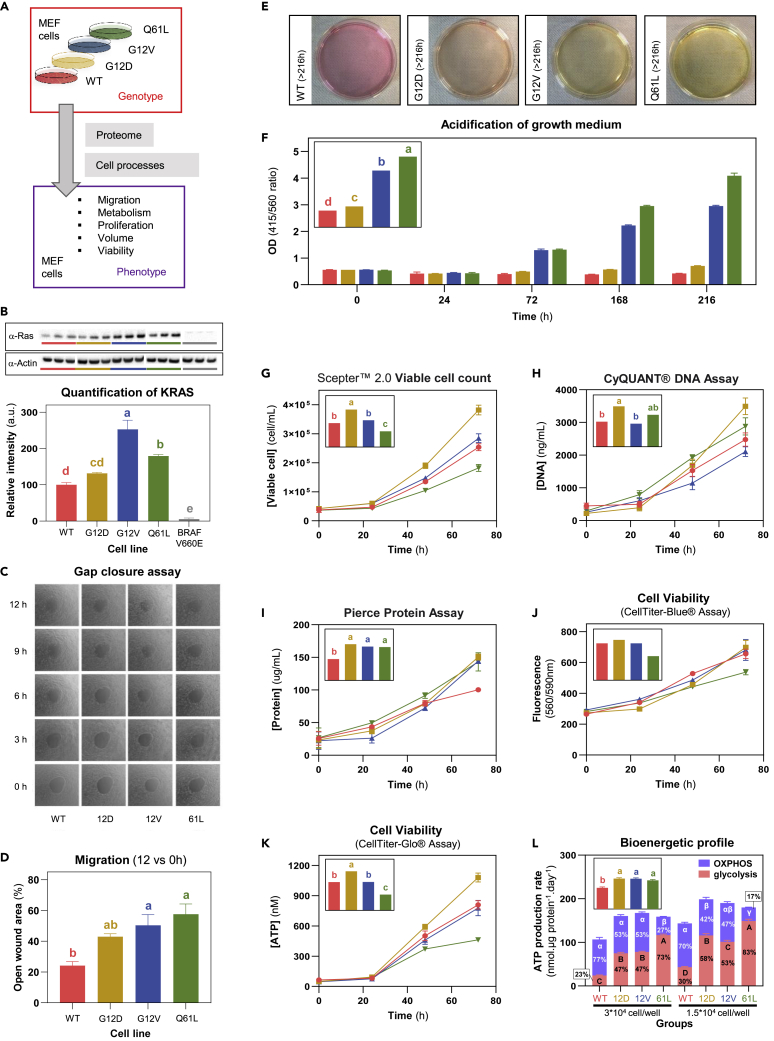
Workflow to analyze the genotype-phenotype relation in RASless mouse embryonic fibroblast (MEF) cell lines and phenotypic data in MEF cells (A) Schematic workflow of MEF cell lines with different genetic backgrounds analyzed in this work. (B) KRAS mutant protein levels obtained by Western blot analysis of lysates of RASless MEF cell lines expressing KRAS WT, different oncogenic KRAS mutants, or BRAF V600E (as negative control for KRAS expression). To obtain nine biological replicates, MEF cell lines were grown for three consecutive weeks with similar cell densities before cell lysis at three different times of the day: 9 a.m., 12 p.m., and 3 p.m.. The western blots show three biological replicates. The bar graph shows the results of nine biological replicates for the abundance of KRAS, normalized by β-actin as loading control. (C) Results of cell migration phenotypes. Representative images of MEF cell lines grown on RadiusTM plates at different time points. (D) The bar graph shows the open wound areas for MEF cell lines at 12 h (three replicates). (E) Power of hydrogen (pH) analysis of MEF cell line growth media. Representative images of MEF culture dishes after 14 days in culture for WT, G12D, G12V, and Q61L MEF cell lines. (F) The bar graph below shows the mean and SEM values of OD 415/560 ratios (triplicates) for MEF cell lines at time 0, 25, 72, 168, and 216 h (each with three biological replicates). The insert represents, for each cell line, the overall least square (LS) mean of all values. (G) Cell proliferation analyzed using the Scepter 2.0 Automated Cell Counter. Time course of viable cells (n = 3). The insert represents, for each cell line, the overall LS of the mean of the proliferation rate calculated between 0 and 72 h. (H) Cell proliferation analyzed using the CyQUANT Assay. Time course of DNA concentration (n = 3). The insert represents, for each cell line, the overall LS of the mean of the proliferation rate calculated between 0 and 72 h. (I) Cell proliferation analyzed using the Pierce™ BCA Protein Assay. Time course of protein concentration (n = 3). The insert represents, for each cell line, the overall LS of the mean of the proliferation rate calculated between 0 and 72 h. (J) Cell viability analyzed using the CellTiter-Blue Assay. Time course of fluorescence intensity (n = 3). The insert represents, for each cell line, the overall LS of the mean of the viability rate calculated between 0 and 72 h. (K) Cell viability analyzed using the CellTiter-Glo Assay. Time course of ATP concentration (n = 3). The insert represents, for each cell line, the overall LS of the mean of the viability rate calculated between 0 and 72 h. (L) Bioenergetic profile measured using the Real-Time ATP-rate assay with a Seahorse XF-analyzer. Total ATP production rate, which is the sum of ATP production through oxidative phosphorylation (blue) and glycolysis (red), was measured for each MEF cell line at 2 cell density and normalized by the protein content of each well measured by Pierce 660 nm Protein Assay (n = 7–8 per group). The insert represents for each cell line, the overall LS mean of the total ATP production rate normalized by the protein content. All statistical analyses displayed are the main effect “cell line” of a two-way ANOVA followed by Tukey’s post-hoc test. Means not sharing any letter are significantly different by the Tukey-test at the 5% level of significance. For example, means labeled by “a” are statistically significantly different from means labeled by “b” (and “c, etc …) but not from means labeled by “ab”.

To analyze the impact of relatively subtle genetic alterations on cell phenotype, we used a panel of genetically engineered MEFs, in which the genes for the three major RAS isoforms (*HRAS*, *KRAS*, and *NRAS*) have been deleted^31^ and that constitutively express human KRAS wild type (WT), KRAS(G12D), KRAS(G12V), or KRAS(Q61L) (Figure 1A). All KRAS mutant cell lines showed elevated KRAS expression levels compared to wild type, with highest levels detected in G12V MEFs (Figure 1B and Table S1). Cells adopted typical fibroblast-like morphologies with branched cytoplasmic processes (extensions) and an elliptical and speckled nucleus (Figure S1C). We observed rounder morphologies for G12V and G12D MEFs. Q61L MEF cells were larger, reaching out with extended cell processes and formed clearer clusters.

Cell migration was analyzed over a time course of 12 h (Figures 1C and 1D and Table S1). All three MEF mutant cell lines, G12D, G12V, and Q61L, migrated significantly less than wild type. Significantly decreased media pH values were observed from day 3 onward for the G12V and Q61L mutant cell lines, and intermediate decreased pH values for the G12D mutant compared to wild type (Figures 1E and 1F and Table S1). These changes in pH are likely caused at least in part by secretion of lactate as a by-product of aerobic glycolysis,^32^ but could also be the result of H+ secretion from ion transporters or CO_2_ from the pentose phosphate pathway.^33^ Proliferation rates (as determined by counting) were highest for G12D MEF cells for both viable and total cells (Table S1, Figures 1G and S2A). However, viable cells were larger for WT and Q61L cells (∼4 pL) compared to G12D and G12V cells (∼3 pL) (Figure S2B). Hence, additional methods for measuring cell proliferation were used. Using the CyQUANT Assay based on DNA content, we measured highest proliferation rates for the G12D mutant, while Q61L and G12V were similar to WT cells (Table S1; Figures 1H and S4A). Using the Pierce™ BCA Protein Assay, we found higher proliferation rates for all mutants compared to wild type (Table S1 and Figure 1I). Proliferation based on packed cell volume increase during 75 h was highest for the G12D and G12V MEF cells compared to WT and Q61L cells (Figure S2C). In line with cells being larger for WT and Q61L, the protein content was highest for WT and Q61L compared to G12D and G12V (Figure S2D). Cell viability was assayed based on the ability of cells to reduce a redox dye into a fluorescent end product (CellTiter-Blue Assay) and based on ATP content (CellTiter-Glo Assay) (Table S1; Figures 1J, 1K, S2E, S2F, and S4B). Viability was highest for G12D, while those of G12V and Q61L MEF cells were lower and more similar to WT. The different patterns of results arising from different indices of proliferation likely arise at least in part from variation in cell size and metabolic state as well as protein content and ploidy.

Seahorse metabolic analysis was performed on all cell lines at two cell densities with similar results at both densities (Figures 1L and S3). There is a marked increase in ATP production rates per ng protein for all the mutant cell lines. This is also associated with an increase shift toward glycolysis that is especially marked in Q61L. Extracellular acidification data show a similar pattern to that seen using pH measurements (Figure 1L) with rates (mpH/min/ug protein) of 3.19, 7.33, 7.82, and 10.84 and 4.97, 10.89, 9.81, and 13.33 for WT, G12D, G12V, and Q61L at the two plating densities (3∗10^4^ and 1.5∗10^4^). In all instances, Q61L has the highest acidification rate.

Finally, we measured uptake and secretion fluxes (normalized to protein content) of key cellular metabolites. Rate of glucose uptake and lactate release show similar patterns (Figures S2G and S2H), with values greater in G12D and Q61L compared to WT and G12V (albeit not significant for glucose uptake). The increase in glucose uptake and lactate release is particularly clear for Q61L when data are normalized per cell (Figures S4C and S4D). The rate of glutamine uptake was significantly lower in Q61L compared to WT while glutamate release rate is similar in all cell lines (Figures S2I and S2J and S4E and S4F). In cancer and proliferative cells, glutamine is widely used as a carbon source for macromolecule synthesis, as an energetic substrate to replenish the tricarboxylic acid (TCA) cycle, as a nitrogen source for DNA production, and as a precursor of glutathione to maintain redox homeostasis.^34^ These data confirm the value of these MEF cell lines as a system where slightly different genotypes relate to clearly distinct phenotypes.

### Whole-cell proteome changes in KRAS wild type and mutant MEF cell lines

**Figure 2:**
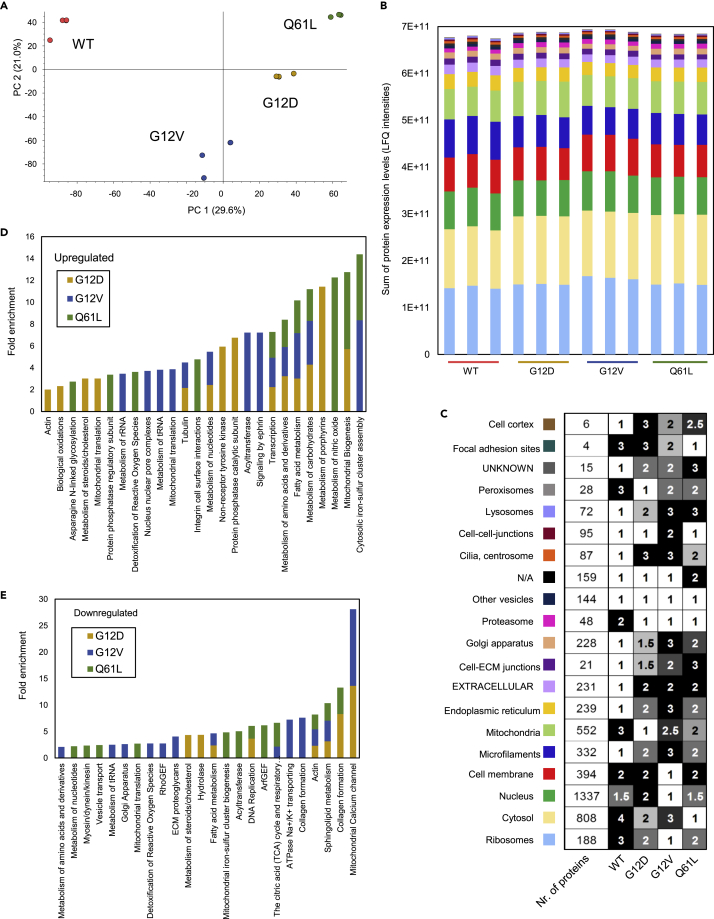
Mass spectrometry-based protein identification in RASless mouse embryonic fibroblast (MEF) cell lines (A) Principal component analysis (PCA) of protein abundances (LFQ intensities) for three biological replicates of KRAS WT and mutant MEF cell lines. (B) Subcellular localization analysis of proteins expressed in KRAS WT and mutant MEF cell lines. Sum of LFQ intensities of proteins belonging to 18 different subcellular localizations as defined in the SysGO database. For each cell line, the three biological replicates are shown. The color code and ordering are identical as in panel (C). (C) Ranking of abundance sums for each subcellular localization for the four MEF cell lines based on the results of one-way ANOVA with Tukey post-hoc tests. Two cell lines separated with a rank value ≥1 have a significantly different (p < 0.05) sum of protein abundances for a specific subcellular localization. The lowest number refers to the highest ranks (i.e. 1 = rank 1 = highest sum of protein abundances). (D) Functional enrichment analysis of upregulated proteins in the G12D, G12V, and Q61L cell lines compared to WT based on the SysGO database. Functional classes that are > 2-fold and significantly enriched are shown (Fisher exact test; p value <0.05). (E) Functional enrichment analysis of downregulated proteins in the G12D, G12V, and Q61L cell lines compared to WT based on the SysGO database. Functional classes that are > 2-fold and significantly enriched are shown (Fisher exact test; p value <0.05).

In-depth proteomic characterization of whole-cell lysates after 48 h growth in fresh medium was performed in triplicates using shot-gun mass spectrometry and label-free quantification (LFQ abundances) (Table S2). Across the 12 samples, the identified peptides were matched to 4896 mouse proteins (filtered for “IsMasterProtein” using the Protein Discoverer tool for processing proteomic data,^35^ with an average of 7.8 peptides per protein used for the identification). Principal component analysis based on the LFQ abundances as a proxy for protein expression levels shows clear alterations in the proteome of WT and mutant MEF cell lines (Figure 2A).

To characterize the protein expression levels in the MEF cell lines on a whole-cell and quantitative level, we used the systematic gene ontology (SysGO) database, which includes (main) subcellular localization and (main) functional information for 19,300 human protein-coding genes,^36^ after matching mouse proteins to their human orthologs (Table S2). We first summed the abundances of proteins belonging to 18 different subcellular localization groups (Figure 2B). When plotting the amounts from high to low abundances, we find that ribosomal proteins comprise the most abundant group of proteins, followed by proteins in the cytosol, nucleus, cell membrane, microfilaments, mitochondria, and the ER. Groups with less abundant proteins are extracellular or associated with cell-ECM junctions, Golgi apparatus, proteasome, other vesicle, cilia/centrosome, cell-cell junctions, lysosomes, peroxisomes, focal adhesion sites, and cell cortex. To compare KRAS WT and mutant cell lines, we next ranked the abundance sums for each subcellular localization from the high (rank 1) to the lower (rank 1.5, 2, 2.5. 3,..) (Figure 2C). For 80% of the subcellular localizations, the wild-type MEF cells ranked either last (rank 4, 3,..) or first (rank 1), suggesting that the respective protein abundances in the mutant cells were either globally downregulated (microfilaments, ER extracellular, cell-ECM junctions, Golgi apparatus, cilia/centrosome, lysosomes, and cell cortex) or upregulated (ribosomes, cytosol, mitochondria, proteasome, peroxisomes, and focal adhesion sites) in specific subcellular localizations. When comparing the mutant cell lines with each other, we find that mitochondria and peroxisomes rank specifically highly (e.g. rank 1, 2) for G12D, ribosomes, and cell membrane for G12V, and cytosol and focal adhesion sites for Q61L MEF cells (Figure 2C).

A protein-centric functional enrichment analysis was performed using SysGO.^36^ The number of proteins differentially expressed compared to WT was highest for Q61L, followed by G12D and G12V (Figures S5A–S5C). Differentially expressed proteins are predominantly linked to metabolism, signaling, gene expression, and proteostasis, but also in cell structure-related categories, such as organelles, cytoskeleton and vesicle transport, and cell adhesion and extracellular matrix (Figures 2D and 2E; Table S2). Upregulated proteins are predominantly enriched in proteins involved in metabolism (e.g. metabolism of carbohydrates, amino acids, fatty acids, mitochondrial biogenesis & translation, and metabolism of tRNA) (Figure 2D). Downregulated proteins are enriched in actin proteins, proteins involved in collagen formation, extracellular matrix proteins, Rho and ArfGEFs, and TCA cycle enzymes (Figure 2E). Neither up- nor downregulated proteins are enriched in the 2260 proteins belonging to a recently reconstructed large-scale networks of Ras-mediated signaling networks (Table S2).^37^

To complement the SysGO analysis, a pathways-centric analysis was performed using the Wikipathway 2021 Human library (Figures S5D, S5E and S5F; Table S2). In line with commonly observed metabolic alterations in cancer cells,^34^^,^^38^ upregulated pathways in all mutants are linked to glycolysis and to the shift of metabolism from oxidative phosphorylation to glucose utilization (Warburg effect). Furthermore, amino acid metabolism and the Cori cycle are upregulated, consistent with the increasing need for amino acids and particularly glutamine by most cancer cells and the need to recycle lactate to glucose through the Cori cycle to reuse it as a fuel to support proliferation.^38^^,^^39^ Additionally, the hypoxia-inducible factor pathway, known to promote angiogenesis and anaerobic metabolism shift in cancer cells,^40^ is upregulated (Figures S5D, S5E, and S5F). Downregulated proteins in all mutant MEF cells are mainly related to extracellular matrix formation, focal adhesions, and cytoskeleton. Some pathways are differentially regulated specifically in some of the mutant cells, such as DNA replication and repair and cholesterol metabolism (downregulated in G12D). The main pathway downregulated in Q61L is oxidative phosphorylation suggesting that this cell line relies on glycolysis for ATP production even more than other mutants.

In summary, protein abundance changes are not restricted to metabolism and signaling, but are found across many functional classes, including those involved in subcellular anatomical structures, such as cytoskeleton, organelles, and cell adhesion. While gene ontology and functional enrichment analyses are informative, there is no direct relationship between abundance and phenotype. Differential expression of proteins comprising these systems provides insights into the capacity for delivery of specific cellular processes but there is also the need to establish what energy is available to drive any of these processes that are active. Given that availability of chemical energy to execute cell work is finite, we also need to capture cell behavior, which we approached in terms of the capacity to deliver energy and the machinery in place to convert this energy into cell work.

### EnerSysGO, a database of “energy-independent” and energy-requiring proteins

**Figure 3:**
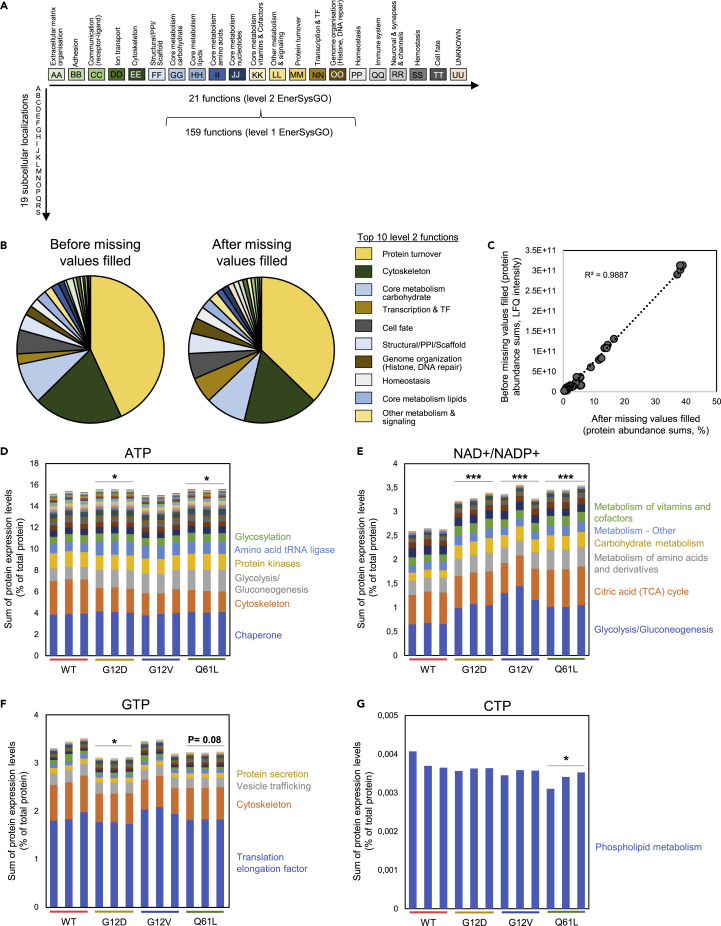
Analysis of energy-requiring processes (A) Schematic representation of the EnerSysGO database. EnerSysGO contains 19,300 protein-coding genes that are assigned to one of 159 functions (level 1) that can be further grouped into 21 functions (level 2). Further, each protein is assigned to one of 19 subcellular localizations. This results in a matrix-like structure, where proteins can be grouped across functions or across subcellular localizations. (B) Sum of protein abundances in 21 EnerSysGO groups before and after missing values filled using the ComPleteROT pipeline for MEF WT cells. (C) Correlation of protein expression sums across 21 EnerSysGO groups before and after missing values filled using the ComPleteROT pipeline for MEF WT cells. (D) Sum of protein abundances of all ATP-requiring enzymes after missing values filled on triplicates separately for MEF WT and mutant cells. The EnerSysGO classes with highest protein abundance sums are indicated on the right side of the bar plots. (E) Sum of protein abundances of all NAD+/NADP + -requiring enzymes after missing values filled on triplicates separately for MEF WT and mutant cells. The EnerSysGO classes with highest protein abundance sums are indicated on the right side of the bar plots. (F) Sum of protein abundances of all GTP-requiring enzymes after missing values filled on triplicates separately for MEF WT and mutant cells. The EnerSysGO classes with highest protein abundance sums are indicated on the right side of the bar plots. (G) Sum of protein abundances of all CTP-requiring enzymes after missing values filled on triplicates separately for MEF WT and mutant cells. The EnerSysGO class with highest protein abundance sums are indicated on the right side of the bar plot. Graphs D-|G were analyzed using one-way ANOVA followed by Dunett’s post-hoc tests. ∗: p < 0.05, ∗∗: p < 0.01, ∗∗∗: p < 0.001 for each mutant versus WT. The EnerSysGO class with highest protein abundance sums is indicated on the right side of the bar plot.

The “demand” side of our whole-cell model analyzes the “fuel” (i.e. energy/ATP/GTP, other high-energy phosphate, or reducing equivalent)-requiring proteins and processes in the cell. We therefore wanted to clearly distinguish proteins that require “fuel” (e.g. tRNA ligases that consume ATP) as opposed to proteins that are required “structure” for delivering a process (e.g. ribosomal structural proteins that do not require ATP to function, once assembled). Building on our recently developed SysGO database,^36^ we created the EnerSysGO database (Table S3). EnerSysGO contains annotations for 19,300 proteins, organized as a matrix of 19 dominant subcellular localizations and 159 main functional classes (level 1) that can be further grouped into 21 functional classes (level 2) (Figure 3A). Clearly, individual proteins may reside in multiple locations and subserve multiple functions but the goal here is to simplify. Distinct subsets of level 1 processes require high-energy phosphates. Examples for such classes are “Transporting ATPases” (e.g. ATP-binding cassette family), “Amino acid tRNA ligase” (e.g. GARS1, Glycine--tRNA ligase), “ATPase Proteasome” (e.g. AAA ATPases such the 26S proteasome), “Serine threonine kinase” (e.g. RAF kinase proteins), and “GTPase RAS Superfamily” (e.g. Ras, Rab, Arf, or Rac family members).

One of the limitations of label-free shotgun proteomics is that the datasets contain a significant number of missing values. However, particularly in the context of considering a system as a whole, it is desirable to have quantitative data for all proteins—at least estimates of approximate order of magnitude. Here, we developed the ComPleteROT (complete proteins) computational pipeline as a systematic solution to the missing values problem (see STAR Methods). ComPleteROT integrates two types of assumptions/information: (1) that proteins in similar functional classes are expressed at approximately similar levels. Indeed, protein abundance averages across different functional SysGO classes obtained from a deep-coverage mass spectrometry dataset of 29 human tissues^13^ show significantly different averages comparing groups of functionally similar proteins (Figure S6); (2) that proteins can be classified into tissue general and tissue specific expressed proteins (if a tissue general expressed protein is not found in an experiment, the assumption is that it should be present and that we predict the missing value). Using information regarding tissue specificity from the Human Protein Atlas database (https://www.proteinatlas.org/), ComPleteROT assigns either average or minimal expression values for a missing protein obtained from other proteins detected in the same functional group (see STAR Methods). Applying ComPleteROT to our proteomics data in the four MEF cell lines shows that the sum of abundances in 21 EnerSysGO groups before and after missing values filled correlate very strongly (Figures 3B and 3C). In the following analyses, we consistently work with the MEF datasets where the missing values have been filled.

In Figures 3D–3G and S7, summary data for key functional classes are shown for each of the cell lines and comparisons between mutant and WT expression levels are given. As might be expected for tumor-associated mutations, carbohydrate metabolism is increased in all mutant cell lines. Various processes associated with protein synthesis and turnover is also increased. Conversely, and reflecting cells’ capacity to shift energy expenditure between different processes, cytoskeletal proteins, and those related to lipid metabolism are decreased. In some instances, there are distinct differences between the mutant lines. In conclusion, there are distinct differences in the energy-consuming protein landscape between cell lines. This led to the question as to whether or not there were supply differences.

### Predicted capacity of MEF cells to generate ATP

To model the maximum rate of ATP synthesis, we adopted the well-established approach of flux balance analysis (FBA; see STAR Methods). This involves the solution of steady-state fluxes that are constrained by upper and lower bounds and the optimization of a so-called objective function, in this instance, the generation of ATP. There are two classes of bounds to consider. The first are those related to reactions that are internal to the model. The second are those constraints imposed by the exchange of metabolites external to the model, for instance in tissue culture media. The upper and lower bounds of each internal reaction flux were set as a function of the enzyme or transporter abundance. The relationship between abundance and flux is approximately linear in the presence of saturating substrate but using a simple linear relationship did not generate a plausible solution, probably reflecting the fact that the concentration of many substrates may be limiting. We therefore explored the impact of a compression transformation that led to progressive saturation of flux bound with increasing protein abundance as a possible approach to improving the biological fidelity of the model. Increasing levels of compression, while keeping the flux of the reaction with the highest flux constant, has the effect of making low-abundance enzymes and transporters relatively more impactful in the determination of the final set of flux bounds. The next constraint was to impose boundaries on rates of metabolite uptake. We used two approaches. First, we made the simplification of assuming that relative maximum uptake rates were proportional to the molar concentration of each metabolite within the MEF cell culture medium. Second, we used measured fluxes for glucose, glutamate, glutamine, and lactate and scaled the other media metabolites involved in exchange reactions in the same proportion to glucose as they are in DMEM medium. We then explored the impact of varying the ratio of overall metabolite availability to the level of bounds set for transporters and enzymes from protein abundances.

**Figure 4:**
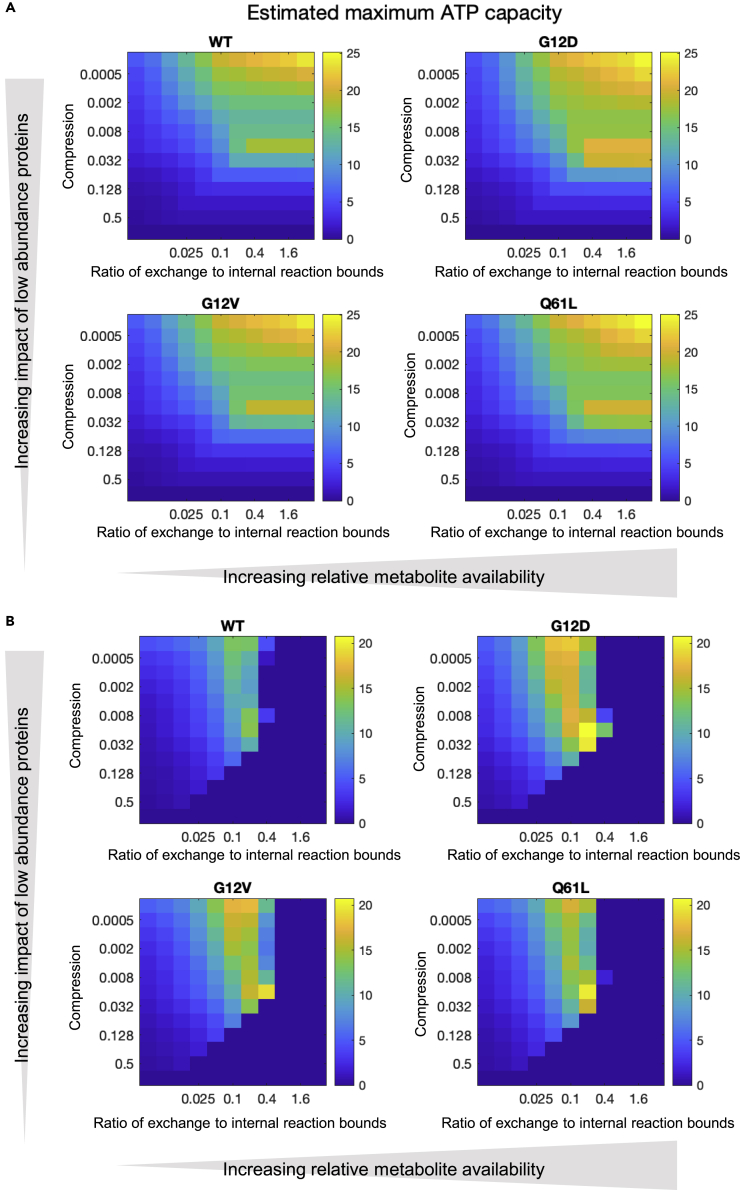
Results of FBA analysis for ATP generation of metabolic models in the four MEF cell lines FBA results for ATP generation expressed as a 2D parameter sweep heatmap. On the “x axis”, the ratio of magnitude of exchange reaction lower bound (which determines the maximum availability of metabolite in the media) to “internal” reaction bounds determined by enzyme or transporter abundance increases from left to right. On the “y axis”, the bottom strip represents a linear mapping of protein expression level to magnitude of upper and/or lower bound for reactions and transport processes where a gene product is defined in Recon3D model. Moving toward the top of the figure the degree there is progressive compression (or saturation) of the relationship between protein abundance and maximum flux such that proteins expressed at low levels have a relatively greater magnitude of upper or lower flux bound than would be the case with a linear relationship. The units are arbitrary and the data a function of both the fitted metabolite uptake and the optimized internal metabolic network. (A) Results of simulations that use exchange reaction bounds estimated in terms of relative abundances within DMEM for WT, G12D, G12V, and Q61L. (B) Results of simulations that use measured values for glucose and glutamine uptake and lactate and glutamate efflux as exchange reaction bounds for these metabolites. WT, G12D, G12V, and Q61L.

By plotting the FBA solution at differing degrees of compression (y axis) and relative metabolite abundance using the DMEM-derived exchange fluxes (x axis), we constructed 2D heatmaps where each square represents a particular combination of saturation and metabolite abundance. Figure S8A shows predicted uptake of glucose when DMEM composition is used to determine substrate availability. There is an interesting peak toward the right of the figure (where the ratio of substrate availability: expression is relatively high) at a compression level of around 0.016. In Figure S8B, glucose uptake is shown when measured exchange rates for glucose, glutamine, glutamate, and lactate are used (Figures S2G–S2J), scaled to the wild-type glucose exchange reaction bound of 25 to assist in comparison with Figure S8A. Toward the right and bottom right portions of the figure, no solution can be found, indicting implausible parameterization. Figures 4A and 4B show similar plots for maximum ATP production as predicted by the GEM model. As would be expected, maximum ATP production is seen toward the right of the figure, where substrate availability in relation to protein expression constraints is higher. Toward the top of the figure, compression of expression levels is higher and so enzymes and transporters expressed at low levels become less constraining. The peak at around 0.016 compression with high metabolite availability is again seen and is a curious property of the metabolic network illustrating the importance of relative contribution of different sets of proteins. Differences between the cell lines are subtle, but maximum ATP supply capacity is seen in G12D and all mutant lines appear able to generate more ATP than WT (see Table 1 for values at a single point on the 2D map). An important property of the metabolic network is the molar ratio of glucose uptake to maximum ATP production (Figure S9 and Equation 1).

(Equation 1) RatioATP/glucose(asinFigureS9)=estimatedmaximumATPflux/estimatedglucoseuptakeflux

**Table 1:** Numerical data from location on 2D heatmaps corresponding to a metabolite availability to internal reaction constraint ratio of 0.05 and a compression value of 0.016

	ATP (arbitrary units)	ATP (pmol ng^−1^ day^−1^)		Net Protein synthesis rate (day ^−1^)	
	DMEM Model	RW Model	Measured	RW Model	Measured
WT	239	183	187	0.135	0.326
G12D	451	399	269	1.102	0.979
G12V	256	297	268	0.673	0.651
Q61L	383	329	254	0.791	0.741

In the RW (“Real-world”) model, measured metabolite uptake rates are used.

**Figure 5:**
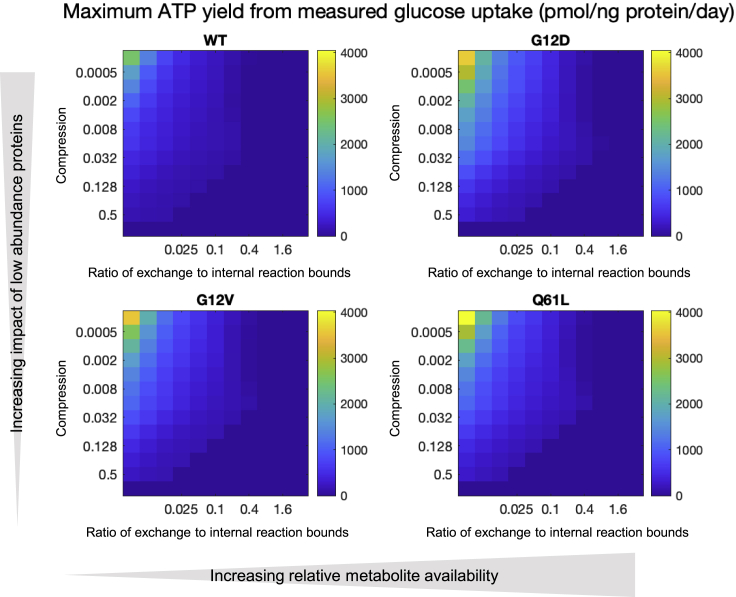
Results of FBA analysis for ATP generation with real glucose uptake rates of metabolic models in the four MEF cell lines FBA results for ATP generation expressed as a 2D parameter sweep heatmap similar as in Figure 4, but instead of normalized glucose uptake rates, actual uptake rates are used (pmol/ng protein/day). Results of simulations that use measured values for glucose and glutamine uptake and lactate and glutamate efflux as exchange reaction bounds but scaled in the same way as for the DMEM plots, that is availability of metabolites in the culture medium relative to enzyme and transporter abundances increases from left to right, for WT, G12D, G12V, and Q61L.

Note that although this is normalized to glucose uptake, other available metabolites in the media (such as amino acids) contribute to total ATP production. The high ratio in the top left hand corner of the plots reflects very low glucose availability relative to protein abundance constraints combined with a high level of expression compression which, again, releases constraints otherwise imposed by enzymes and transporters expressed at low levels. A further refinement is to use, instead of fitted glucose uptake rates normalized to DMEM composition, actual uptake rates (Figure 5 and Equation 2).

(Equation 2) $$ATP\;from\;measured\;glucose\;uptake\;\left(mol.time^{-1}\right)=ATP/glucose\;*\;measured\;glucose\;uptake\;\left(mol.time^{-1}\right)$$

Here, we have measured glucose uptake in pmol.ng protein^−1^.day^−1^. As we are multiplying this by the dimensionless ratio of ATP generation per glucose uptake, the maximal rate of ATP production has the same units. It now becomes possible to compare the 2D solution space (Figure 5) with the results of the Seahorse analysis (Figure 1L). The FBA solution closest to the measured average rate of ATP production for WT cells is with a metabolic availability to protein abundance constraint ratio of 0.05 and a compression factor of 0.016. Taking this as a calibration point for the other plots, it is possible to readout predictions from other analyses. The central columns in Table 1 give predicted and measured values for a range of parameters. The pattern of ATP production rates between cell lines is different, with the model predicting a particularly low rate for G12V but all mutant cells lines show the expected increase in rate.

In summary, the model predicts that all the mutant cell lines demonstrate increased capacity to synthetize ATP with G12D having the highest capacity and this mirrors the Seahorse results (Figure 1L).

### Distribution of available ATP flux to different cellular processes

Attempting to predict the activity of multiple cellular executive processes using FBA is a challenge as optimizing an objective function around one process denies all the others if there is competition between processes for given substrates. Composite objective functions can be created, for instance for maximizing biomass. This approach has proved effective in prokaryotes and synthetic biology settings^41^^,^^42^ but less so in mammalian systems^43^ and using biomass as the objective function showed a difference between WT and mutant lines but did not clearly discriminate between mutant lines in a way that predicted observed data (data not shown).

The EnerSysGO classification was used to generate a table of summed enzyme abundances for each EnerSysGO class at each EnerSysGO location (Figure S7 and Table S3) and that identified those enzymes and transporters that were ATPases, requiring ATP. Some clear patterns emerge. In all the mutant cell lines, there is, as would be expected, increased glycolysis and pentose phosphate pathway enzyme abundance, whereas the TCA cycle is diminished (group GG). There is increased metabolism of amino acids and processes related to protein synthesis and turnover (cf. within group II) but in contrast, ATP-consuming reactions related to cytoskeleton (group EE) are markedly reduced as might have been predicted from the cell migration data (Figures 1C and 1D).

We sought to map available ATP as estimated from FBA across all the ATP-consuming processes. For consistency, we compressed the ATP-consuming enzyme and transporter abundance in the same way as for the metabolism enzymes and transporters. In addition, where individual proteins combine to form complexes or multiple enzymes catalyzed single reactions, the same “minsum” rules that were employed for the FBA were adopted. Where kcat data were available, the product of corrected abundance and kcat was used to estimate the “share” of ATP directed to that reaction. A mean kcat value was used where data were not available.

To further explore protein synthesis, we took the amino acid-tRNA ligase group (within group MM) as an indicator of rate of protein synthesis. This energy-demanding step in protein synthesis consumes two ATP molecules for every amino acid (AA) bound to a tRNA for subsequent addition to a peptide chain. Taking the average molecular weight of an amino acid as 110 Da, the estimated maximum capacity for ATP generation, and the percentage of ATP-consuming reactions represented by the abundance of AA-tRNA ligases corrected with the protein rules and kcat, it is possible to estimate rates of protein production and compare with measured values. Taking the calibration point determined by the Seahorse data (ie compression of 0.016 and substrate to internal constraint ratio of 0.05), it was possible to predict rates of total protein synthesis. We directly measured net protein synthesis, and so to compare the net and total rates, we need to estimate the amount of protein synthesis that contributes to protein turnover alone. The range of protein turnover rates is very large^44^ but we have assumed an average turnover rate of 0.5/day. Table 1 shows there is reasonable correspondence between predicted and measured net protein synthesis rates. Taking a compression level of 0.016 as an estimate of an overall informative level of compression, it is possible to use the same approach for all the other classes of ATP and GTPase. In this instance, we have used abundance values corrected for kcat where available. By then taking a metabolite availability index of 0.05 from the Seahorse calibration, the maximum rate of ATP available to support ATPases and GTPases can be estimated for each cell line (from Figure 5). These data combine to generate a predicted whole-cell energy budget. This is shown in Table 2. It is a challenge to map the activity of each of these processes to phenotype but some general conclusions are possible. There is a general increase in all cell processes related to cell proliferation and the magnitude of this increase is enhanced by taking into account increased metabolic rate, suggesting that an energy-corrected interpretation of protein abundances may be more informative than abundances alone (as seen in Figure S7). The calibration of the model also makes it possible to explore specific estimated fluxes of other parameters. This is done by multiplying the estimated flux from the flux balance analysis by the ratio of measured glucose uptake to the predicted uptake for the model. For instance, the exchange rates for protons (81.6, 74.0, 61.2, and 93.6 for WT, G12D, G12V, and Q61L, respectively) can be seen to have a similar pattern to the measured ECAR data with Q61L being the highest, but WT is higher than G12D and G12V which is distinct from the measured data. Exchange of other metabolites and the fact that buffering is not taken into account probably contribute to the discrepancy.

**Table 2:** Energy budget for EnerSysGO classes contributing to 90% of the total energy demand (ATP + GTP)

	% of total expression (corrected for kcat at compression of 0.016)				ATP flux (pmol/ng protein/day)			
EnerSysGO class (short name; see Table S3)	WT	G12D	G12V	Q61L	WT	G12D	G12V	Q61L
058_Nucelotide metabolism (ATP)	1.90	1.95	1.96	1.94	24.56	55.24	40.81	45.10
011_Cytoskeleton (ATP)	1.49	1.46	1.45	1.45	19.22	41.25	30.25	33.88
116_Glycosylation (ATP)	1.14	1.12	1.13	1.17	14.79	31.86	23.48	27.37
096_Chaperone (ATP)	1.02	1.09	1.07	1.07	13.13	30.76	22.27	25.04
084_Amino acid tRNA ligase (ATP)	0.97	1.03	1.03	1.01	12.51	29.16	21.35	23.47
012_Cytoskeleton (GTP)	0.72	0.68	0.70	0.71	9.31	19.32	14.54	16.58
107_Protein kinases (ATP)	0.44	0.41	0.43	0.44	5.67	11.61	8.97	10.22
106_Vesicle trafficking (GTP)	0.44	0.43	0.42	0.39	5.64	12.18	8.73	9.16
027_Carbohydrate metabolism-other (ATP)	0.41	0.42	0.43	0.42	5.25	11.88	8.86	9.71
076_Metabolism of mRNA (ATP)	0.41	0.40	0.43	0.40	5.35	11.20	8.89	9.24
064_Metabolism of vitamins and cofactors (ATP)	0.37	0.40	0.38	0.36	4.79	11.25	7.85	8.29
016_Glycolysis and Gluconeogenesis (ATP)	0.31	0.32	0.32	0.32	4.05	9.14	6.75	7.41
131_Chromatin organization (ATP)	0.31	0.29	0.30	0.33	4.06	8.35	6.30	7.59
006_Receptor kinase (ATP)	0.35	0.28	0.32	0.28	4.49	7.80	6.67	6.55
009_Transporter ions (ATP)	0.29	0.26	0.30	0.30	3.77	7.33	6.28	6.94
020_Citric acid and TCA cycle (ATP)	0.27	0.27	0.27	0.27	3.43	7.59	5.61	6.22
136_DNA replication (ATP)	0.28	0.24	0.28	0.28	3.66	6.80	5.74	6.52
102_Protein secretion (ATP)	0.21	0.22	0.22	0.22	2.69	6.20	4.57	5.23
068_Transporter other (ATP)	0.21	0.23	0.22	0.21	2.70	6.46	4.57	4.88
103_Protein secretion (GTP)	0.23	0.21	0.20	0.21	2.92	6.03	4.18	4.80
042_Phospholipid metabolism (ATP)	0.18	0.18	0.19	0.19	2.26	5.20	3.88	4.37
070_G protein (GTP)	0.21	0.15	0.17	0.17	2.72	4.12	3.64	3.92
052_Metabolism of amino acids (ATP)	0.16	0.17	0.18	0.16	2.05	4.79	3.66	3.67
155_Ras GTPases (GTP)	0.18	0.15	0.15	0.15	2.30	4.36	3.13	3.55
081_Metabolism of tRNA (ATP)	0.14	0.16	0.16	0.15	1.80	4.59	3.32	3.56
118_Ubiquitination-activation-E1s (ATP)	0.15	0.16	0.15	0.15	1.91	4.49	3.03	3.50

## Discussion

There are increasingly abundant datasets available that provide rich descriptions of protein and mRNA expression, but it remains challenging to integrate these data to predict cell behavior in a quantitative or even semiquantitative way. Given the fundamental biological significance of energetics, we have explored in this study the proposition that there is value in formulating a whole-cell energy model that is anchored in energy production and utilization. For many systems, energy production is a key constraint and the available chemical energy must be optimally distributed between multiple cellular processes to support cell survival, homeostasis, and core functionality.^3^ A complete description of the dynamics of cellular energetics is far from current reach but our intent was to explore the extent to which a simple approach might be informative. The approach we took was to simplify both supply, using flux balance analysis to estimate capacity to generate ATP, and demand, by distributing available ATP between ATP-consuming enzymes and transporters.

We have explored the potential of such an approach by characterizing in detail the behavior of mouse embryonic fibroblasts with different Ras genetics and testing the predictive power of a proteomic dataset. The behavior of the different mutants was distinct, both in relation to their phenotype and their metabolism and ultimately, metabolism determines phenotype. The various Ras mutations will impact differentially on total GTP loading and associated signal transduction pathway activation, but here we focused on signaling/transcription-induced downstream protein changes. Figure S7 summarizes a wide array of cellular processes and how they differ by cell line. Distinct patterns emerge, which in some instances global increase or decrease expression in mutant lines in comparison to wild type and others where notably G12D and Q61L are more elevated than G12V.

For both supply and demand sides of the model, there was a challenge as to how to handle missing values which arise from proteomic datasets. Gaps arising from technical issues could have created critical failures in the metabolic model and biased the ATPase analysis. We used a composite approach that incorporated grouping of proteins by process and the ubiquity of expression as defined in the Human Protein Atlas. True negatives will have been lost but in the current study the summary statistics of the imputed and raw datasets were similar and the former were used for all subsequent analyses. This approach may be of value in other studies.

### Limitations of the study

The complexity of the genome, the multiplicity of function of many gene products, and incompleteness of knowledge present challenges in the development of gene ontologies. Here, we have chosen to create an ontology from the perspective of cell energetics, that acknowledges spatial compartments as well as cell processes and for the latter clearly distinguishes processes that consume ATP. There are inevitable simplifications, but the advantage is being able to get a snapshot approximation of how the cell appears to be utilizing energy.

FBA provides a computationally efficient technique for predicting metabolic fluxes where upper and lower bounds of reaction fluxes are estimated and an objective function is optimized. There are many disadvantages of FBA and one of the challenges is that it does not take into account enzyme kinetics or substrate/product concentrations; thus it is not surprising that, for instance, predicted glucose uptake and measured glucose uptake are different. Other challenges of FBA include balancing the relationship between external metabolite availability and protein or gene expression-determined reaction bounds, as well as establishing the relationship between protein or gene expression and the magnitude of flux bounds. We have adopted an empirical approach, exploring how the output of the objective function changes with different parameters. While somewhat arbitrary, this offers a way forward. In the future, more objective approaches to parameter setting will be required. There are many refinements of FBA which could be incorporated into the work flow presented here. Additional refinements will include a shift toward more energy-based modeling^45^^,^^46^ and addressing what is apparent from the current study, namely that even on the supply side, objective functions steer the simulation into a specific solution that can be misleading with, for instance, a potential over-emphasis on oxidative phosphorylation.

The EnerSysGO analysis confirmed that protein synthesis is a major metabolic demand on the cell and the whole-cell energy model was used to estimate the ATP requirements for a key part of this process and cross reference to measured protein synthesis rates. Despite the large number of assumptions made, there is a reasonable correspondence between measured and predicted protein synthesis rates (Table 1). With further enhancements that consider enzyme kinetics and metabolite concentrations as well as the compartmentalized spatial organization of the cell, we believe that the approach taken in this study will prove to be a powerful method for the prediction of cell behavior from “omic” datasets. A key attribute is its quantitative nature. This facilitates the use of statistics and the detection of relatively subtle shifts of cell processes in the context of what the whole-cell is capable of, in other words, the overall energy constraints of the cell.

In summary, we have a proposed a supply and demand framework for the modeling of cell metabolism. By taking capacity to generate ATP into account, the interpretation of the relative abundances of proteins associated with various processes can be enhanced.

## STAR★Methods

### Key resources table

**Table:** 

REAGENT or RESOURCE	SOURCE	IDENTIFIER
**Antibodies**		

β- Actin (13E5) Rabbit mAb	Cell Signaling	RRID: AB_4970
Recombinant Anti-Ras antibody [EP1125Y]	Abcam	RRID: AB_ab52939

**Critical commercial assays**		

Radius™ 24-Well Cell Migration Assay	Cell Biolabs, Inc.	# CBA-125
CyQUANT Cell Proliferation Assays	ThermoFisher Scientific	# C7026
Pierce™ BCA Protein Assay	ThermoFisher Scientific	# 23225
CellTiter-Blue® Cell Viability Assay	Promega	# G8081
CellTiter-Glo® Luminescent Cell Viability Assay	Promega	# G9241
Glucose-Glo™ Assay	romega	# J6021
Lactate-Glo™ Assay	Promega	# J5021
Glutamine/Glutamate-Glo™ Assay	Promega	# J8021

**Deposited data**		

Mass spectrometry analysis of MEF cell lines	This paper	MassIVE: MSV000089464; Table S1
Protein expression levels in 29 human tissues	Wang et al., 2019	Table EV1
Tissue-specific expression information	The Human Protein Atlas	https://www.proteinatlas.org/humanproteome/tissue
SysGO database	Luthert and Kiel, 2020	Table S1

**Experimental models: Cell lines**		

RAS-less mouse embryonic fibroblast (MEF) isogenic cell lines transduced with either RAS genes (KRAS 4B wild type, KRAS 4B G12D, KRAS 4B G12V, KRAS 4B Q61L, or constitutively active BRAF (BRAF V600E)	The RAS Initiative at the Frederick National Laboratory	N/A

**Software and algorithms**		

New Matlab code developed in this work	This work	GitHub: https://github.com/pluthert/MEF-metabolism and Zenodo with https://doi.org/10.5281/zenodo.7469975

### Resource availability

#### Lead contact

Further information and requests for resources and reagents should be directed to and will be fulfilled by the lead contact, Christina Kiel (christina.kiel@unipv.it).

#### Materials availability

This study did not generate new unique reagents.

### Experimental model and subject details

RAS-less mouse embryonic fibroblast (MEF) isogenic cell lines,^31^ transduced with either RAS genes (KRAS 4B wild type, KRAS 4B G12D, KRAS 4B G12V, KRAS 4B Q61L, or constitutively active BRAF (BRAF V600E), were obtained from *The RAS Initiative* at the Frederick National Laboratory (https://www.cancer.gov/research/key-initiatives/ras/ras-central/blog/2017/rasless-mefs-drug-screens). All culture incubations were performed in a humidified 37°C and 5% CO2 incubator. Cell lines were cultured in MEF culture medium, containing Dulbecco’s modified Eagle medium (DMEM) high glucose (4500 mg/L glucose, 0.2 mM L-glutamine, Thermo Fisher Scientific), 10% (v/v) heat inactivated fetal bovine serum (FBS) (heat inactivated, Thermo Fisher Scientific), and Blasticidin S HCl (10 mg/mL) (Thermo Fisher Scientific). For conducting phenotypic assays, cells were cultures in growth medium without Blasticidin S HCl.

### Method details

#### Cell lysis and western blotting

MEF cells were lysed using 1x PathScan Cell lysis buffer (Cell Signaling), containing 20 mM Tris-HCl (pH 7.5), 150 mM NaCl, 1 mM disodium EDTA, 1 mM EGTA, 1% Triton, 20 mM sodium pyrophosphate, 25 mM sodium fluoride, 1 mM b-glycerophosphate, 1 mM Na3VO4, 1 μg/mL leupeptin, following the manufacturer’s instructions. Cell lysates were loaded on SDS gels (NuPAGE Bis-Tris Gels from Invitrogen or Criterion Precast Gels from Biorad) and separated by electrophoresis. Gels were then transferred onto nitrocellulose membrane using the iBlot2 system (Invitrogen), and the blots were incubated 1 h at room temperature in TBS, Tween 0.1% + 5% milk. The primary antibody was incubated at 4°C overnight (1:1000 dilution), and the HRP-coupled secondary antibody (1:10,000 dilution) was incubated 1 h at room temperature, both in TBS Tween 0.1% +  0.5% milk. Blots were developed using high sensitivity ECL reagent (Thermo) and visualized using the G:BOX image developer (SYNGENE). Bands were analyzed using ImageJ. The following antibodies were used for Western blotting: β-Actin (#4970 Cell Signaling) and panRas (ab52939 Abcam).

#### Cell migration analysis

Cell migration was assayed using a gap closure assay (Radius™ 24-Well Cell Migration Assay, CELL BIOLABS INC.) following the manufacturer’s instructions. Briefly, each plate well contains a 0.68 mm hydrogel spot where cells cannot attach when seeded. MEF cells were seeded in triplicates with a seeding density of 1.0 × 10^5^ cells per well. Once attachment of the MEF cells was achieved (overnight), the hydrogel was removed to expose the cell-free region. Cell migration/closure was monitored by capturing images with an inverted microscope at 3h, 6h, 9h, and 12 h after removal of the hydrogel. Images were analyzed using CellProfiler™ Cell Image Analysis Software (https://cellprofiler.org/).

#### Assessment of pH in tissue culture medium

Phenol red added to tissue culture media is a visual pH indicator that exhibits a gradual transition from yellow to red over a pH range of 6.2 to 8.2. To assay the pH in the cell culture medium the change in the absorbance spectrum of phenol red at 415 and 560 nm with was used as indicator of the change in pH (https://www.biotek.com/resources/application-notes/using-phenol-red-to-assess-ph-in-tissue-culture-media/). Briefly, cells were seeded in 24-well plates in triplicates with a seeding density of 0.1–0.5 × 10^4^ cells per well. To measure absorbance at different days (using Spectramax M3), 100 μL were transferred into a 96-well plate in triplicates, incubated at 5% CO2 for at least 5min, and spectral reads from 400-600 nm as well as endpoint measurement at 415nm and 560nm were performed. A higher 415/560 nm ratio was indicative of lower pH.

#### Cell proliferation and viable cell volume

For each MEF cell lines, cultures at 80–90% confluency were seeded in 6-well plates (CELLSTAR, Greiner Bio-One) at ∼6 × 10^4^ cells per well in 1.5 mL (∼6 × 10^3^ cell.cm^−2^) and incubated at 37°C and 5% CO_2_. At each time point, culture medium was removed, cells were washed twice with phosphate buffer saline (PBS), detached with 0.5 mL Trypsin-EDTA (Gibco™, cat.no. 25200056) and resuspended in 1 mL culture medium (1.5 mL final cell suspension). Cell concentration and volume were measured directly after the sampling by diluting cell suspension four times in PBS and using Scepter™ 2.0 Automated Cell Counter with 60 μm sensors (Merck Millipore). Both total and viable cell count and volume were recorded.^47^ The whole experiment was repeated three times with two technical replicates.

#### DNA amount with CyQUANT™ assay

Cell proliferation was analyzed using the CyQUANT™ Assay (Thermo Fisher Scientific). For each MEF cell lines, cultures at 80–90% confluency were seeded in 6-well plates (CELLSTAR, Greiner Bio-One) at ∼1 × 10^5^ cells per well in 1.5 mL and incubated at 37°C and 5% CO_2_. At each time point, 100 μL suspension was sampled and processed following the manufacturer’s instructions. The whole experiment was repeated three times with two technical replicates.

#### Pierce™ BCA protein assay

Protein content was analyzed using the Pierce™ BCA Protein Assay (Thermo Fisher Scientific). For each MEF cell lines, cultures at 80–90% confluency were seeded in 6-well plates (CELLSTAR, Greiner Bio-One) at ∼1 × 10^5^ cells per well in 1.5 mL and incubated at 37°C and 5% CO_2_. At each time point, cells were sampled and processed following the manufacturer’s instructions. The whole experiment was repeated three times with two technical replicates. Net protein synthesis rate at 48 hours was calculated by dividing the difference in abundance between 24 and 72 hours and dividing by two days.

#### CellTiter-blue® Cell Viability Assay

The number of viable MEF cells was determined using the CellTiter-Blue® Cell Viability Assay (Promega) following the manufacturer’s instructions. Briefly, cells were seeded in 24-well plates in triplicates with a seeding density of 0.1–0.5 × 10^4^ cells per well and incubated overnight. After 0 h, 24 h, 48 h and 72 h, the CTB reagent (Promega) was directly added to the wells of 96-well plates, mixed carefully, and incubated for 1–4 h in the dark at 37°C. The fluorescence was measured at 560/590 nm (Spectramax M3).

#### Cell viability analysis using CellTiter-Glo

Cell proliferation was analyzed using the CellTiter-GloAssay (Promega). For each MEF cell lines, cultures at 80–90% confluency were seeded in 6-well plates (CELLSTAR, Greiner Bio-One) at ∼1 × 10^5^ cells per well in 1.5 mL and incubated at 37°C and 5% CO_2_. At each time point, cells were sampled and processed following the manufacturer’s instructions. The whole experiment was repeated three times with two technical replicates.

#### Energetic profile analysis using seahorse

Assessment of ATP production arising from glycolysis and oxidative phosphorylation was performed by Seahorse XFp Analyser (Agilent Technologies, Santa Clara, CA, USA) using the Seahorse XFp Real-Time ATP Rate Assay Kit (Agilent Technologies) according to the manufacturer’s instructions. Briefly, MEF cell line WT and mutants were seeded into Agilent Seahorse XFp well plates at two densities: 3∗10^4^ and 1.5∗10^4^ cell/well and eight wells per cell density, in the complete growth medium and were incubated for 24 h at 37°C and 5% CO2. One hour before measurement, the medium was replaced with Seahorse XF Assay Medium supplemented with 2 mM L-glutamine, 1 mM sodium pyruvate and 10 mM glucose after one wash with the same medium. Then, the plates were placed for 1 h at 37°C in a non-CO2 incubator. The XF Real-Time ATP Rate assay template of the Wave Pro 10.0.1 software (Agilent Technologies) was used to run the assay with Oligomycin and Rotenone/Antimycin A injected at final concentrations of 1.5 μM and 0.5 μM, respectively. After the assay, the Seahorse XFp well plates were placed at −20°C before measurement of protein concentration using PierceTM 660 nm Protein Assay Reagent (Thermo Fisher) after cell lysis using PathScan® Sandwich ELISA Lysis buffer (Cell Signalling Technology). Protein content was used to normalise the oxygen consumption rate (OCR) and the extracellular acidification rate (ECAR). Finally, ATP production rate data were obtained with the Seahorse Analytics website (Agilent Technology) using the XF ATP Rate Assay view.

#### Whole-cell lysate MS-based proteomic analyses

The four MEF cell lines (KRAS wildtype and KRAS mutant cell lines G12D, G12V, and Q61L) were analyzed in triplicates (10-cm dishes with 1–2 x 10^6^ cells per dish). MEF cells were lysed in lysis buffer supplemented with 1 X cOmplete protease Inhibitor Cocktail tablets (Sigma) and centrifuge at high speed for 10 min at 4°C. Supernatant were recovered and transferred into a new tube. The protein concentration of cell lysates was determined by using the Pierce^TM^ BCA protein assay (Thermo Fisher Scientific) following the manufacturer instruction/protocol. For each sample, approximately 50 μg of proteins were adjusted in 20 μL of buffer/MS grade water. The same volume of urea (8 M) was added, followed by the double volume of ammonium bicarbonate (NH_4_HCO_3_) and 100 mM of calcium chloride (CaCl_2_). The proteins were reduced in 0.2 M of dithiothreitol (DTT) for 15 min at room temperature followed by 0.4 M of iodoacetamide (IAA) and incubate for 15 min at room temperature in the dark. Magnetic beads were prepared by combining 5 μL of hydrophilic and 5 μL of hydrophobic beads and washed several times in MS grade water. Beads were added in the deepwell plate on the KingFischer Duo Prime as well as the cell lysate and the Trypsin (0.5 μg/μL). In another deepwells 80% ethanol was added for the washing steps. After 4.5 h on the KingFischer, samples were transferred into new tubes, 0.1% formic acid was added, and samples were dried in speed vacuum and stored at −20°C.

At the time of the MS analysis, the aliquots were thawed at room temperature and dissolved in MS grade water. The samples were run on a Thermo Scientific Q Exactive mass spectrometer connected to a Dionex Ultimate 3000 (RSLCnano) chromatography system. Tryptic peptides were resuspended in 0.1% formic acid/2.5% acetic acid. Each sample was loaded onto a fused silica emitter (75 μm ID, pulled using a laser puller (Sutter Instruments P2000), packed with Reprocil Pur C18 (1.9 μm) reverse phase media (Dr Maisch Gmbh) and was separated by an increasing acetonitrile gradient over 120 minutes at a flow rate of 200 nL/min. The gradient details are as follows: From 0-16 min the sample was loaded on to a c18 packed fused silica emitter tip at a flow rate of 350 nL/min with 1% buffer B (using the gradient pump). From 16-120 minutes buffer b was increased from 1-27 % at 200 nL/min, from 120-122 minutes buffer B was increased from 27-95% at 200 nL/min, from 122–132.5 minutes the column was washed at 95% buffer B, from 132.5-133 minutes buffer B was decreased from 95-1% and the flow increases from 200-350 nL/min. Buffer B (80% acetonitrile, 19.9% water, 0.1% formic acid) and buffer A (98% water, 1.9% acetonitrile, 0.1% formic acid) used LC-MS grade solvents. The total length of the gradient is 133 minutes, but the mass spectrometer does not acquire data for 13 min due to the loading step and dead volume hence the chromatogram duration is 120 min. The mass spectrometer was operated in positive ion mode with a capillary temperature of 320°C, and with a potential of 2300 V applied to the frit. All data was acquired with the mass spectrometer operating in automatic data dependent acquisition (DDA) switching mode. A high resolution (70,000) MS scan (350–1600 m/z) was performed to select the 12 most intense ions prior to MS/MS analysis using higher energy collision dissociation (HCD). Other MS1 parameters used included an automatic gain control (AGC) target of 3e6 and a maximum injection time (IT) 60ms.^48^ MS2 setting used were: resolution 17500; AGC target 5e4; maximum IT 250 ms; isolation window 1.6 m/z; isolation offset 0 m/z; fixed first mass 100 m/z; normalised collision energy (N) CE 27; minimum AGC 5e3; exclude unassigned and 1; preferred peptide match; exclude isotopes and dynamic exclusion was set to 45 s^48^ All mass spectrometry raw data was deposited in MassIVE (https://massive.ucsd.edu/ProteoSAFe/static/massive.jsp) with accession number MSV000089464.

#### MS data processing

The Proteome Discoverer software platform (Thermo Scientific, version 2.3) was used to process mass spectrometry raw files and searches were conducted against the *Mus musculus* complete reference proteome (Uniprot, released in March, 2019), using Sequest HT as search engine. The match-between-runs feature was enabled using default settings. Database searches were performed with the following parameters: Fixed Mod: cysteine carbamidomethylation; Variable Mods: methionine oxidation; Trypsin/P digest enzyme (maximum two missed cleavages); Precursor and fragment mass tolerance was set to 10 ppm and 0.8 Da respectively. Identified peptides and proteins were filtered using a False Discovery Rate (FDR) set at 1% and a Peptide Spectrum Match (PSM) set at 1%.

#### Enrichment analysis using the SysGO database

The 4986 proteins detected by mass spectrometry were first translated to human gene IDs using the "Mouse Human ID conversion" using g:Profiler (https://biit.cs.ut.ee/gprofiler/convert). Except for 159 genes, all could be matched to any of the 19300 human protein-coding genes in the SysGO database.^36^ Proteins present in the MEF cell lines were matched to 43 out of the 47 subcellular localization groups (“SysGO localization—set 1”). The 43 groups were further merged into 19 classes, namely “Ribosomes”, “Cytosol”, “Nucleus” (merging of Nucleus, Nuclear envelope, Nucleoplasm, Chromatin, Nucleoli fibrillar center, Nuclear speckles, Nuclear bodies, and Necleoli), “Cell membrane”, “Microfilaments” (merging of Microfilaments (Actin), Intermediate filaments (Keratin, filaments), and Microtubules), “Mitochondria”, “Endoplasmic reticulum”, “Extracellular”, “Cell-ECM junctions”, “Golgi apparatus”, “Proteasome”, “Other vesicles” (merging of Melanosomes, Endosomes, Outer segments, and Lipid droplets), “Cilia, centrosome” (merging of Cilia, Centrosome, Microtubule organising centre, Midbody, and Mitotic spindle), “Cell-cell-junctions”, “Lysosomes”, “Peroxisomes”, “Focal adhesion sites”, “Cell cortex”, and “UNKNOWN”. In addition to subcellular localisation annotations, the SysGO database contains (main) functional annotation for each protein-coding gene (321 classes; “SysGO—set 1”), of which 132 are related to signaling functions (Luthert and Kiel, 2020). For visualization purposes, some classes are merged, resulting in a total of 58 groups (“SysGO—set 2”).^36^ This can be further reduced to 15 groups (“SysGO—set 3”)^36^ that correspond to the classes of “Signaling”, “Metabolism”, “Protein translation, folding, modification and degradation”, “Transcription”, “Unknown”, “Cytoskeleton”, “Organelles”, “Other”, “Immune system and Inflammation”, “Chromatin organization and DNA repair”, “Neuronal System, synapses, channels”, “ECM organization”, “Cell junction and adhesion”, “Developmental”, and “DNA Replication”.

#### Enrichment analysis using wikipathway 2021

In addition to the system analysis, the whole-cell lysate has been analysed using a pathway-centric approach. The most significantly differentially expressed proteins between WT and the three mutants were identified using 1% FDR cut-off (see quantification and statistical analysis). Venn diagrams were then performed using the list of all differentially expressed, under-expressed or overexpressed proteins in the three mutants using BioVenn tool (https://www.biovenn.nl/). This software provided the number and the list of under- and overexpressed proteins specific to each mutant or shared between two or three mutants. Finally, from these lists of proteins, a pathway enrichment analysis has been performed with Enrichr tool using Wikipathway 2021 Human library. Pathways were ranked using the p-value of the Fisher’s exact test.^49^

#### EnerSysGO database

Based on the 19300 human protein-coding genes of the SysGO databases, EnerSysGO annotates the 1968 proteins that use high-energy phosphates (ATP, GTP, CTP) or reduction equivalents (NAD+, NADP+) in the reactions that they are catalyzing. Examples for ATP classes are “Transporting ATPases” such as ATP binding cassette family or “Amino acid tRNA ligase” (e.g. GARS1, Glycine--tRNA ligase). EnerSysGO also includes an improved annotation of functional classes (based on the SysGO database^36^) and a representation as matrix. On the x-axis of the matrix EnerSysGO includes 159 level 1 functions that include information of the subset of 1968 energy/reduction equivalent requiring enzymes. For example, there are four classes of glycoslysis/gluconeogeneis: 015_Glycolysis / Gluconeogenesis, 015_Glycolysis / Gluconeogenesis ATP, 015_Glycolysis / Gluconeogenesis GTP, and 015_Glycolysis / Gluconeogenesis NAD. Level 1 functional classes are combined to 21 level 2 functional classes, which are “AA_Extracellular matrix organization“, “BB_Adhesion“, “CC_Communication (receptor-ligand) “, “DD_Ion transport“, “EE_Cytoskeleton“, “FF_Structural/PPI/Scaffold“, “GG_Core metabolism carbohydrate“, “HH_Core metabolism lipids“, “II_Core metabolism amino acids“, “JJ_Core metabolism nucleotides“, “KK_Core metabolism vitamins&Cofactors“, “LL_Other metabolism&signaling“, “MM_Protein turnover“, “NN_Transcription&TF“, “OO_Genome organisation (Histone, DNA repair)“, “PP_Homeostasis“, “QQ_Immune system“, “RR_Neuronal&synapses&channels“, “SS_Hemostasis“, “TT_Cell fate“, and “UU_UNKNOWN“. On the y-axis of the matrix EnerSysGO includes 19 subcellular localisations, which are “A_Extracellular“, “B_Cell ECM junctions“, “C_Cell cell junctions“, “D_Cell membrane“, “E_Cytosol“, “F_Ribosomes“, “G_Endoplasmic reticulum“, “H_Golgi apparatus“, “I_Mitochondria“, “J_Lysosomes“, “K_Peroxisomes“, “L_Other vesicles“, “M_Microfilaments (Actin, Tubulin) “, “N_Cilia, centrosome“, “O_Cell Cortex“, “P_Focal adhesion sites“, “Q_Proteasome“, “R_Nucleus“, and “S_Unknown“. The EnerSysGO database is available on GitHub (ExpressionData.xlsx).

#### Computing missing values using ComPleteROT

The number of proteins that can be detected by mass spectrometry is limited and therefore out of ∼19300 proteins a certain percentage of proteins can be missing. To compute estimates for abundances of missing values, we developed ComPleteROT, which has two basic principles as assumption: (1): assume that proteins in similar functional classes (using the SysGO database) are in the same expression order of magnitude; (2) use knowledge of HumanProteinAtlas (tissue general vs tissue specific proteins). In brief, missing values were replaced with the mean expression level of related proteins. For each missing value the script identifies the associated SysGO functional class (level 1, level 2, or level 3) and the Human Protein Alas category (“Detected in all“, “Detected in many“, “Detected in some“, “Detected in single“, “Not detected”, and “#N/A”). Based on the Human Protein Alas category, values are assigned from average or minimum expression values of all detected proteins in the respective SysGO functional class, with the following rules: “Detected in all - > Average”, “Detected in many - > Minimum”, “Detected in some - > Minimum”, “Detected in single - > Minimum”, “Not detected - > Zero”, and “#N/A - > Minimum”. The Matlab script is available on Github.

#### Flux balance analysis

The metabolic model is based on Recon3D model,^9^ which consists of 10,600 reactions, where 5938 of them contain known proteins/enzymes, and 2797 metabolites across seven compartments (cytosol, mitochondria, peroxisome, Golgi apparatus, endoplasmic reticulum, nucleus, and lysosome). We chose Recon3D model rather than mouse genome-scale models as the MEF cell system expresses human KRAS variants and we were interested in exploring metabolism in the context of human cancer. The COBRA Toolbox was used with Matlab 2020b and the Gurobi 9.1 solver. A small number of minor modifications to the core model were made. Scripts and additional functions are available on GitHub. Identifiers for proteins in the Recon3D model were converted to human gene symbols (the most recent gene symbols/ names were used; see SysGO database.^36^ Some reactions in Recon3D model contain ‘gene-reaction rules’, which specify if proteins contain more than one subunit (‘AND’ logic) or if isoforms or similar protein family members can catalyse the same reaction (‘OR’ logic). Here, we summed abundances for proteins that are part of the same reaction with an ‘OR’ rule; we took the minimum abundance for proteins being part of complexes (‘AND’ rule), the so-called ‘minsum’ approach. The resulting corrected abundances were used to set the upper and lower bounds of the reactions. A linear mapping of abundance to bound magnitude appeared to result in a limited number of very highly expressed proteins dominating and a large number of very low fluxes so a saturating function analogous to the Michaelis-Menten equation was adopted with varying estimates of the ‘Km’ term and normalizing to ‘Vmax’. For reactions without known enzymes, upper and lower bounds were not constrained beyond the model defaults. Exchange reactions were initially scaled based on DMEM (growth medium for MEF cell lines) composition (mM concentration in DMEM from 0 to 25). None of the various refinements of FBA including enzyme constraint, determination of thermodynamic feasibility or removal of infeasible reactions were employed. The objective function was set to maximize ATP supply. Analyses were run with differing ratios of exchange reaction to ‘internal’ protein abundance – set bounds and with differing degrees of compression of the relationship between protein abundance and magnitude of upper and lower flux bounds. The results of each analysis were then plotted as a heat map. Where appropriate results were normalized to ng measured protein per day.

Where measured external fluxes were incorporated into the flux balance model (“Real-world”, RW model) all of the unmeasured flux bounds were kept constant and two normalization steps were used to integrate the measured flux rates into the model. First, they were scaled so that the wild-type glucose flux bound was set to −25, that is the same in the default ‘DMEM’ set of constraints. There was still the potential for imbalance between measured glucose flux and the rest of the model so a further normalization was carried out setting the glucose flux bound for all cells lines to −25 and scaling the other metabolites accordingly. The model then generates a set of fluxes that are in a molar relationship to each other. To translate these fluxes back to explore the relationship with other measured parameters, it is necessary to ‘calibrate’ against a measured flux, in this context typically glucose uptake. Full details of how the scaling was carried out can be found in the code on GitHub.

#### ATP demand model

A list of ATP–requiring reactions and abundance of associated proteins was generated using ComPleteROT for missing values and a similar approach to complexes and multiple isoforms as for enzymes in Recon3D model (see above). In addition, the same saturation function relating abundance to biological activity (see above) was employed. Subgroups were constructed using the EnerSysGo database which made it possible to determine the percentage of ATP consuming enzyme for given processes and / or location in relation to the total abundance of ATP consuming reactions. Protein synthesis rates were estimated by taking advantage of the stoichiometry of the amino acid tRNA ligase reaction. Each amino acid incorporated into a polypeptide requires 2 ATPs for this step. After correction for abundance compression and kcat the abundance of the tRNA ligases, as a percentage of total ATPase corrected abundance, was used to determine the percentage of ATP flux that could be directed to protein synthesis. Taking the average molecular weight of an amino acid as 110 Da the rate of protein production could be estimated.

#### Biomass proliferation and metabolite turnover

For each MEF cell lines, cultures at 80–90% confluency seeded in three 6-well plates (CELLSTAR, Greiner Bio-One) at ∼6 ∗ 10^4^ cell.well-1 in 1.5 mL (∼6 ∗ 10^3^ cell.cm^−2^) and incubated at 37°C and 5% CO_2_. The three plates corresponded to three days of analysis with three sampling time per days (24 h, 48 h, 72 h and, for each time point, 3 h before and after) and with two biological replicate per sampling time. At each time point, culture medium was removed, cells were wash twice with phosphate buffer saline (PBS), detached with 0.5 mL Trypsin-EDTA (Gibco™, cat.no. 25200056) and resuspended in 1 mL culture medium (1.5 mL final cell suspension). This suspension was used to measure packed cell volume (PCV) in duplicate for each well. At 0 h, 24 h, 48 h, and 72 h time points, before discarding the medium, 500 μL were sampled for metabolite assay. In addition, from the remaining cell suspension, 25 μL, 125 μL and 100 μL were sampled for DNA analysis, ATP analysis and cell counting, respectively. Samples were stored at −20°C until analysis, either directly or after centrifugation at 500 g for 5 min and supernatant discarding for DNA. Cell concertation and volume were measured directly after the sampling by diluting cell suspension four times in PBS and using Scepter™ 2.0 Automated Cell Counter with 60 μm sensors (Merck Millipore). Both total and viable cell count and volume were recorded.^47^ The whole experiment was repeated three times.

#### Protein content assessment

For each MEF cell line, cultures at 80–90% confluency were seeded in six 60-mm dishes (Greiner Bio-One) at ∼1.5 ∗ 10^5^ cell.well^−1^ in 4 mL (∼7 ∗ 10^3^ cell.cm^−2^) and incubated at 37°C and 5% CO2. At 0 h, 24 h, 48 h and 72 h, the cells of two dishes were resuspended in 4 mL Trypsin-EDTA + culture medium and two samples of 1 mL per dishes were spined at 500 g for 5 min, the supernatant discarded, and cell pellets were stored at −20°C for protein analysis. With the remaining suspension, 1 mL were used to perform PCV analysis, and a cell count was measured with Scepter™ 2.0 as described earlier. The whole experiment was repeated twice.

#### Packed cell volume analysis

PCV was measured by transferring 600 to 1000 μL cell suspension in PCV tubes (Techno Plastic Products) and by centrifugating 1 min at 2500 g at room temperature as recommended by Stettler et al.^50^ The volume of the cell pellet packed in the capillary of the tube, was measured using “easy read” Measuring Device (Techno Plastic Products). Pellet volume was then normalized to the suspension volume to obtain a PCV value in μL cell/ mL suspension.

#### Metabolite and biochemical compound assays

Glucose, lactate, glutamine, and glutamate concentration were measured using Glucose-Glo™ Assay, Lactate-Glo™ Assay and Glutamine/Glutamate-Glo™ Assay (Promega), respectively. Culture medium dilution of 1/500, 1/100 and 1/50 in PBS were used to measure glucose, lactate, and glutamine/glutamate concentration, respectively. Cell ATP concentration was measured using CellTiter-Glo® Luminescent Cell Viability Assay (Promega), following the manufacturer’s instructions. Cell DNA content was measured using CyQUANT™ Cell Proliferation Assay (Invitrogen) by resuspending cell pellets in 250 μL CyQUANTTM GR dye/cell-lysis buffer. Finally, cell protein content was measured by resuspending cell pellets in PathScan® Sandwich ELISA Lysis Buffer (Cell Signalling Technology) then by using Pierce™ 660 nm Protein Assay Reagent (Thermo Scientific™) following the manufacturer’s instructions. For all these assay, absorbance, fluorescence, and luminescence were read with SpectraMax® M3 plate reader (Molecular Devices).

#### Normalization and metabolic flux calculations

For biomass, biochemical and metabolite parameters, to limit the variability between experimental repeats, for each cell line, all values of each experiment were normalized by the average value of the three experiments. After normalization, for all parameters an overall flux of production, uptake, or release (in quantity.mL-1.day-1) was calculated by taking the difference between the value at day d and the average value at day d-1 and dividing it by d-(d-1). For biochemical and metabolite parameters, the overall flux was then divided by the average total cell concentration at day d and d-1 to obtain a rate of production per cell (quantity.cell-1.day-1). Finally, by dividing the rate of production per cell by the average protein content per cell (overall protein concentration divided by total cell concentration) at day d and d-1, a rate of production normalized by protein content is obtained (in quantity.ng protein-1.day-1). Fluxes predicted by FBA were determined around 48 h after seeding, so the average of the daily flux rates from 24 h to 72 h were used for comparison.

### Quantification and statistical analysis

Values are presented as mean ± SEM unless indicated otherwise. For metabolic parameters, data were analyzed using two-way ANOVA followed by Tukey’s post-hoc test after validation of residual normality using Shapiro-Wilk’s test and variance homogeneity using Spearman’s test for heteroscedasticity. If one of the two conditions was not validated (p-value <0.05) a log transformation of the data was performed before the ANOVA. Correlation between two variables was analyzed using Pearson’s test. *P*-values <0.05 were considered significant. For whole-cell lysate analysis, differential protein expression was log transformed and comparison with WT was performed using multiple Student’s tests with 1% False Discovery Rate using method of Benjamini et al.^51^ All statistical analyses were performed using GraphPad Prism® 8.0 software.

## Data Availability

• Mass spectrometry data have been deposited at the MassIVE repository (https://massive.ucsd.edu/ProteoSAFe/static/massive.jsp) and are publicly available. Accession numbers are listed in the key resources table. • This paper analyzes existing, publicly available data. These accession numbers for the datasets are listed in the key resources table. • All original code has been deposited at GitHub and is publicly available as of the date of publication. DOIs are listed in the key resources table. • Any additional information required to reproduce this work is available from the lead contact upon request.

## Supplemental information

Document S1. Figures S1–S9

Table S1. Phenotype data in RASless mouse embryonic fibroblast (MEF) cell lines

Table S2. Mass spectrometry based proteomics data in RASless mouse embryonic fibroblast (MEF) cell lines and SysGO analysis

Table S3. EnerSysGO analysis
