# Supplementary material for: Whole-cell energy modeling reveals quantitative changes of predicted energy flows in RAS mutant cancer cell lines
Source: iScience. 2023 Jan 5;26(2):105931. doi: 10.1016/j.isci.2023.105931 (PMC9874014; doi:10.1016/j.isci.2023.105931)
Supplement: Document S1. Figures S1–S9 [file mmc1.pdf]

iScience, Volume *26*

## Supplemental information

# Whole-cell energy modeling reveals quantitative changes of predicted energy flows in RAS mutant cancer cell lines

**Thomas Sevrin, Lisa Strasser, Camille Ternet, Philipp Junk, Miriam Caffarini, Stella Prins, Cian D'Arcy, Simona Catozzi, Giorgio Oliviero, Kieran Wynne, Christina Kiel, and Philip J. Luthert**

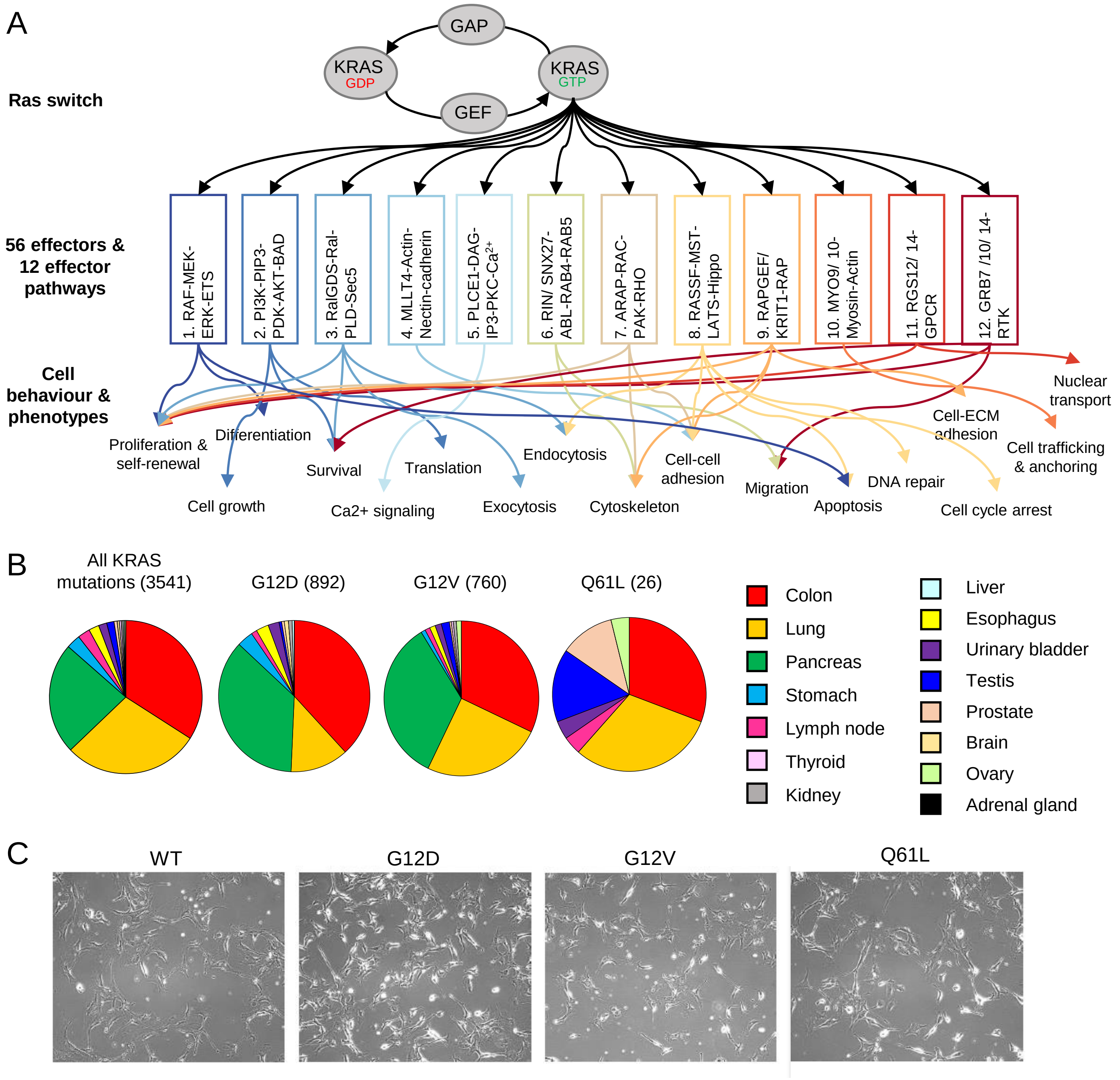


**Figure S1. KRAS as a central switch controlling cell behaviour, KRAS mutation frequencies in cancers of different primary tissue sites, and morphologies of RAS-less MEF cell lines used in this study. Related to Figure 1.**

(A) Ras as a central switch controlling cell behaviour and phenotypes. Schematic diagram of the molecular switch of Ras family GTPases. The Ras GTPases cycle between GDP-bound inactive and GTP-bound active conformations. In the active form, Ras proteins interact with 56 effector proteins that converge onto 12 effector pathways controlling cell behaviour and phenotypes.

(B) KRAS mutation frequencies in cancers of different primary tissue sites. Distribution of KRAS mutation-containing cancers according to the tissue type for all 3541 KRAS cancer mutations (see legend). The number of samples are shown in brackets. The most frequent mutations are found at the codons G12 (75.6 %), G13 (10.5 %), and Q61 (6.1 %). The KRAS allelic distribution across all cancers is G12D (30.0 %), G12V (22.3 %), G12C (13.5 %), G13D (8.8 %), followed by mutations with lower frequency (G12A, G12S, Q61H, Q61L, Q61R). All data were obtained from the cBioPortal database (https://www.cbioportal.org/).

(C) Morphologies of RAS-less MEF cell lines transduced with either wildtype or mutant KRAS genes (G12D, G12V or Q61L) at 48 h.

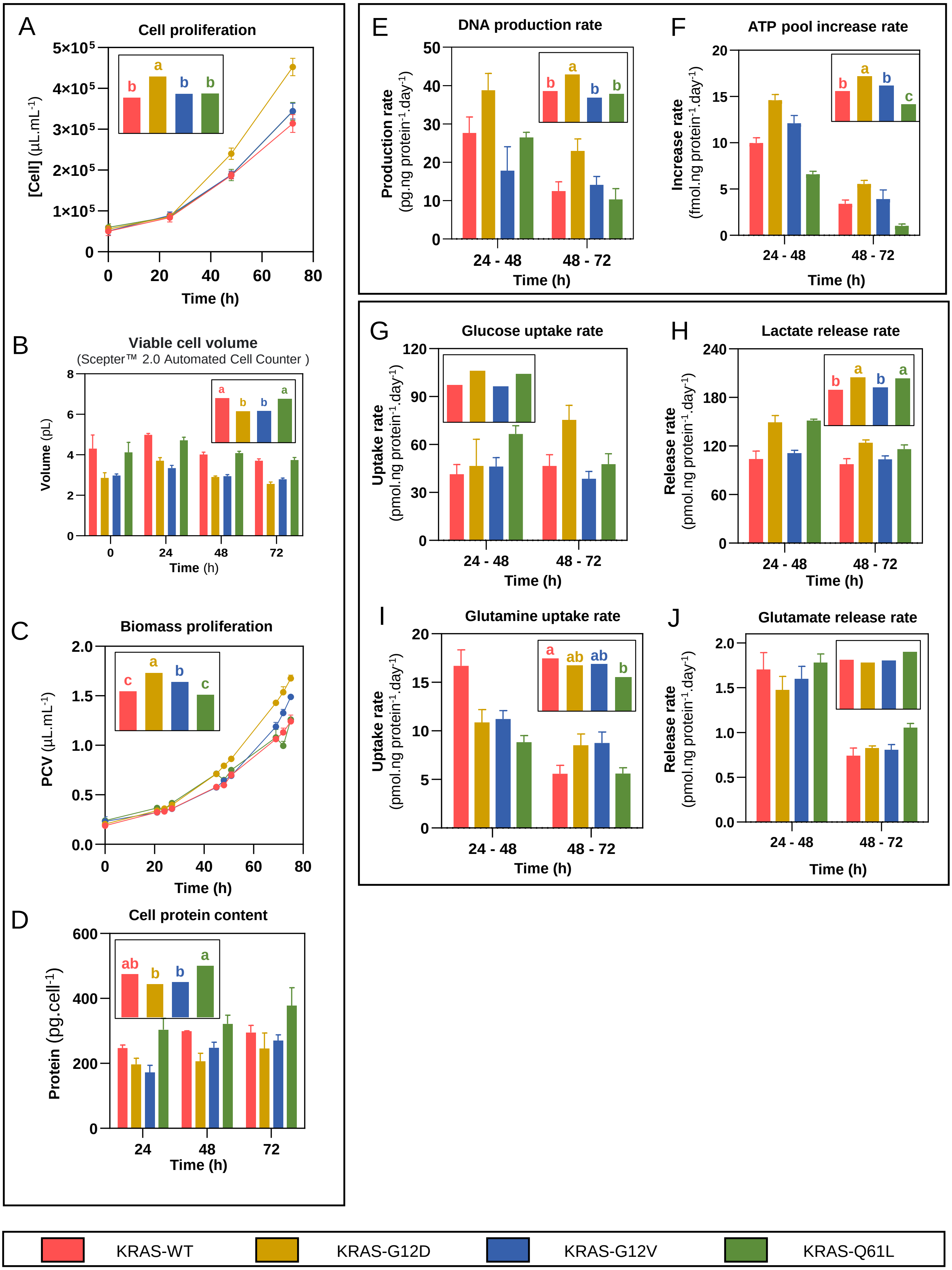

A
Cell proliferation
[Cell] (µL.mL⁻¹)
Time (h)
B
Viable cell volume
(Scepter™ 2.0 Automated Cell Counter )
Volume (pL)
Time (h)
C
Biomass proliferation
PCV (µL.mL⁻¹)
Time (h)
D
Cell protein content
Protein (pg.cell⁻¹)
Time (h)
E
DNA production rate
Production rate
(pg.ng protein⁻¹.day⁻¹)
Time (h)
F
ATP pool increase rate
Increase rate
(fmol.ng protein⁻¹.day⁻¹)
Time (h)
G
Glucose uptake rate
Uptake rate
(pmol.ng protein⁻¹.day⁻¹)
Time (h)
H
Lactate release rate
Release rate
(pmol.ng protein⁻¹.day⁻¹)
Time (h)
I
Glutamine uptake rate
Uptake rate
(pmol.ng protein⁻¹.day⁻¹)
Time (h)
J
Glutamate release rate
Release rate
(pmol.ng protein⁻¹.day⁻¹)
Time (h)
KRAS-WT
KRAS-G12D
KRAS-G12V
KRAS-Q61L

**Figure S2. Biomass proliferation and metabolite production of MEF cell expressing KRAS wild-type or mutant. Related to Figure 1.**

(A) Cell proliferation measured by recording the total cell concentration in each well after cell resuspension.

(B) Viable cell volume analysed using Scepter™ 2.0 Automated Cell Counter at different time points (n=3). The insert represents, for each cell line, the overall least square (LS) mean of all values.

(C) Biomass proliferation measured by packed cell volume (PCV) analysis.

(D) Average protein content of a single cell obtained by dividing the total protein content by the total number of cells.

(E-J) Rate of increase of metabolites normalized by the protein content for DNA production (E), ATP pool of the cells (F), glucose uptake (G), lactate release (H), glutamine uptake (I), and glutamate release (J).

For panels A and C, the inserts represent, for each cell line, the overall least square (LS) of the mean of the proliferation rate calculated between 24h and 72h (values in µL.mL-1.day-1 and cell.mL-1.day-1 for panels A and B, respectively). For panels D to J, the inserts represent, for each cell line, the overall LS mean of the parameter displayed in the panel. Values are mean ± SEM and n=6 (n=3 for panel D only). Statistical analysis displayed are the main effect “cell line” of a two-way ANOVA followed by Tukey’s post-hoc test. Different letters indicate significant difference ($p < 0.05$) between two cell lines.

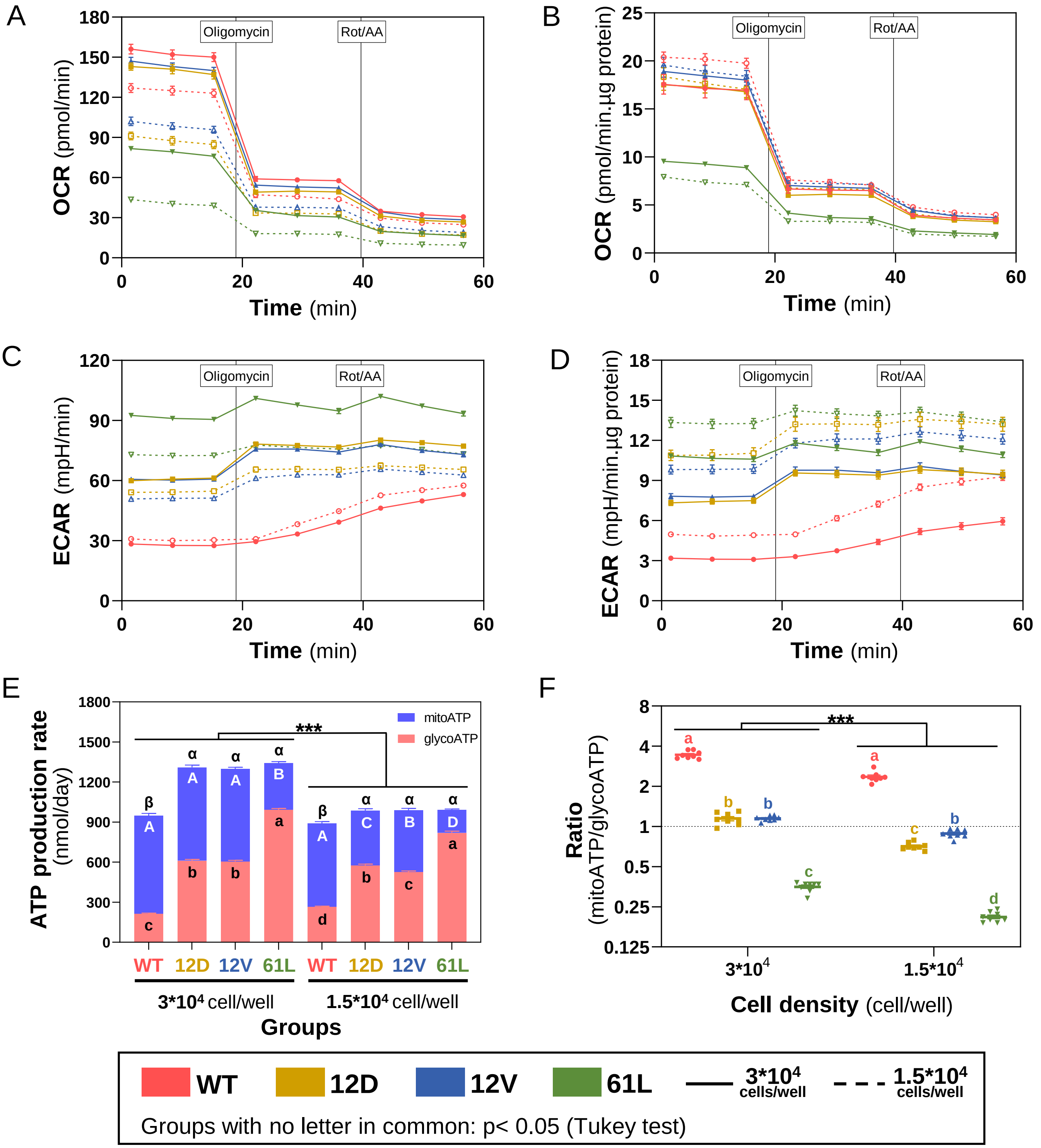


**Figure S3. ATP-rate assay of MEF cells at 2 different cell densities using Seahorse. Related to Figure 1.** Oxygen consumption rate (A) of the whole well or (B) normalized by protein content. Extracellular acidification rate (C) of the whole well or (D) normalized by protein content. (E) Total ATP production rate of the whole well which is the sum of ATP produced by oxidative phosphorylation (mitoATP) and by glycolysis (glycoATP). Results were calculated in nmol/day to be consistent with values of ATP flux obtain with the FBA model. (F) ATP rate ratio was obtained by dividing mitoATP by glycoATP. Values are the mean ± SEM of 7-8 replicate wells. ATP production rate and ATP rate ratio were analysed by 2-way ANOVAs followed by Tukey's post-hoc tests. For the main effect "cell density": *** : p-value <0.001. In each cell density cell line means not sharing any letter in common are significantly different (p<0.05). For ATP production lowercase letters are used to display differences in glycoATP, uppercase letters for differences in mitoATP, and Greek letter for differences in total ATP production.

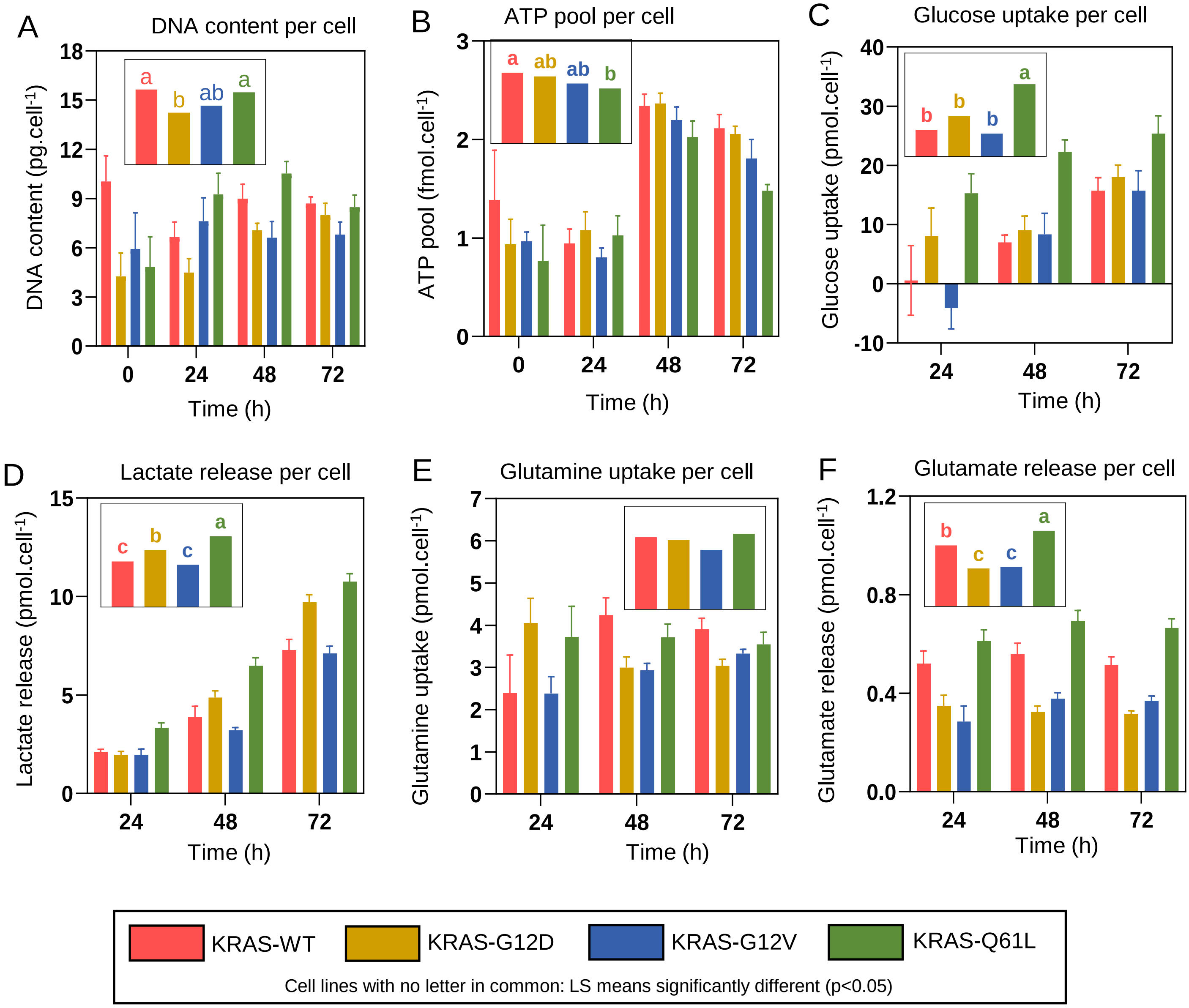


**Figure S4: Single cell biochemical compound and metabolite production MEF cell expressing KRAS wide-type or mutant. Related to Figure 1.**

(A) DNA and (B) ATP content of a single cell obtained by normalizing each day the concentration value by the total cell concentration. (C) Glucose uptake, (D) lactate release, (E) glutamine uptake, and (F) glutamate release of a single cell, obtained by normalizing each day the cumulative uptake/release by the total number of cells. The inserts represent, for each cell line, the overall LS mean of the parameter displayed in the panel. Values are mean ± SEM and n=6. Statistical analysis displayed is the main "cell line" effect of a two-way ANOVA followed by Tukey's *post-hoc* test. Two cell lines that do not share a letter have significantly different LS mean ($p<0.05$).

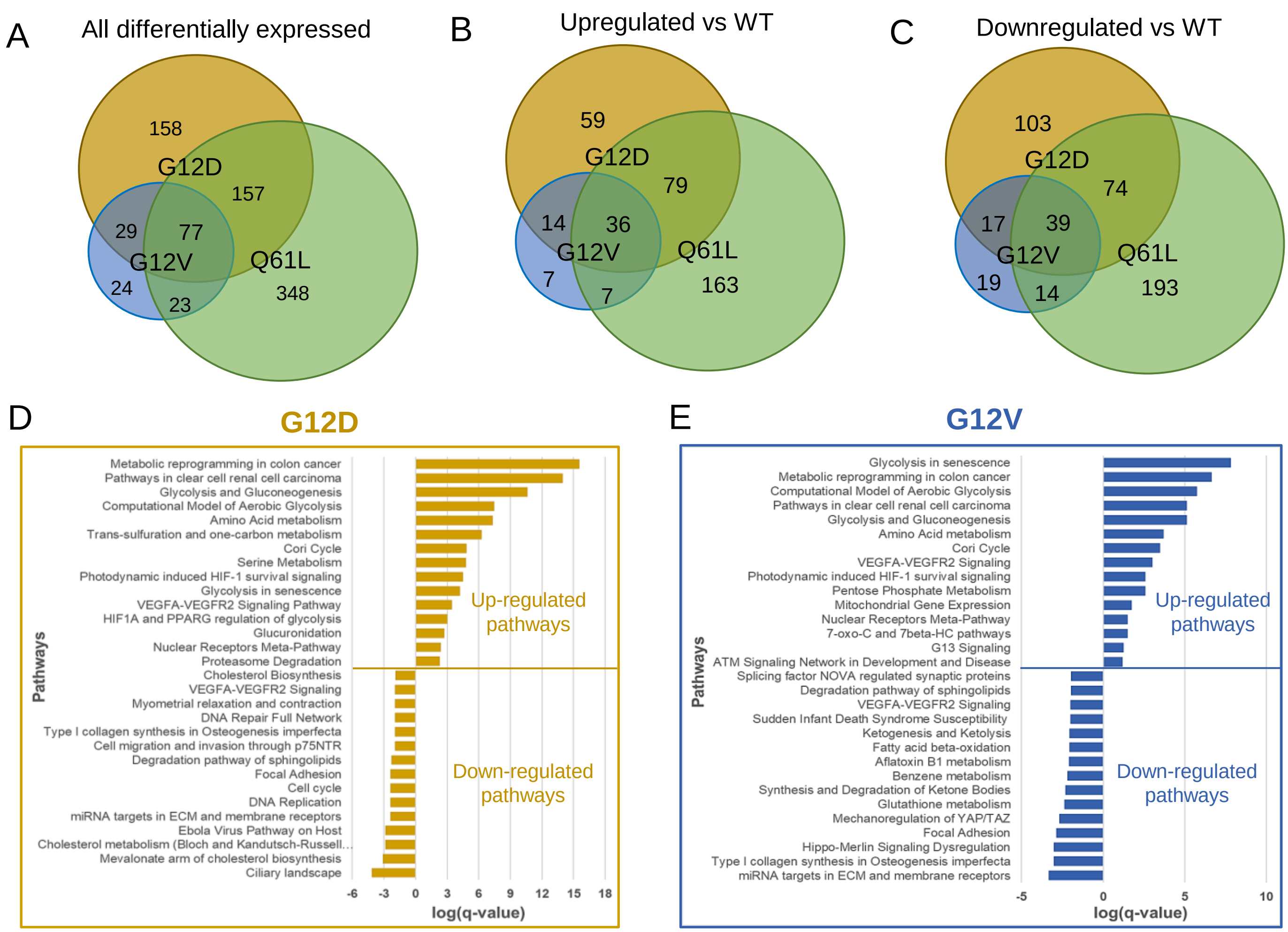


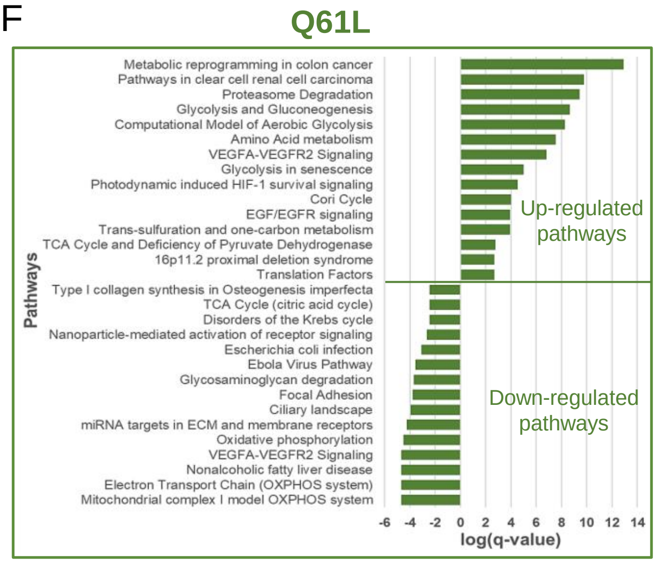


**Figure S5. Pathway-centric enrichment analysis of differentially expressed protein in the three mutant cell lines compared to WT. Related to Figure 2.**

(A)-(C) Venn diagrams of the proteins significantly differentially expressed in the three mutant MEF cell lines compared to WT using multiple t-tests with 1% FDR correction. The Venn diagram was performed using BioVenn software and the color manually adjusted in ppt.

(D-F). Pathway-centric enrichment analysis of differentially expressed protein in the three mutants compared to WT. Enrichment analysis was performed with Enrichr software inputting for each subgroup the list of up or down-regulated genes separately. Enrichment was based on Wikipathway 2021 Human library and pathway ranked by the q-value of Fisher's exact test corrected by 5% FDR. The top 15 up-regulated and down-regulated pathway are displayed with –log(q-value) for up-regulated pathways and log(q-value) for down-regulated pathways (positive and negative values, respectively). The higher the absolute value of the log(q-value) is the closer to 0 is the actual q-value.

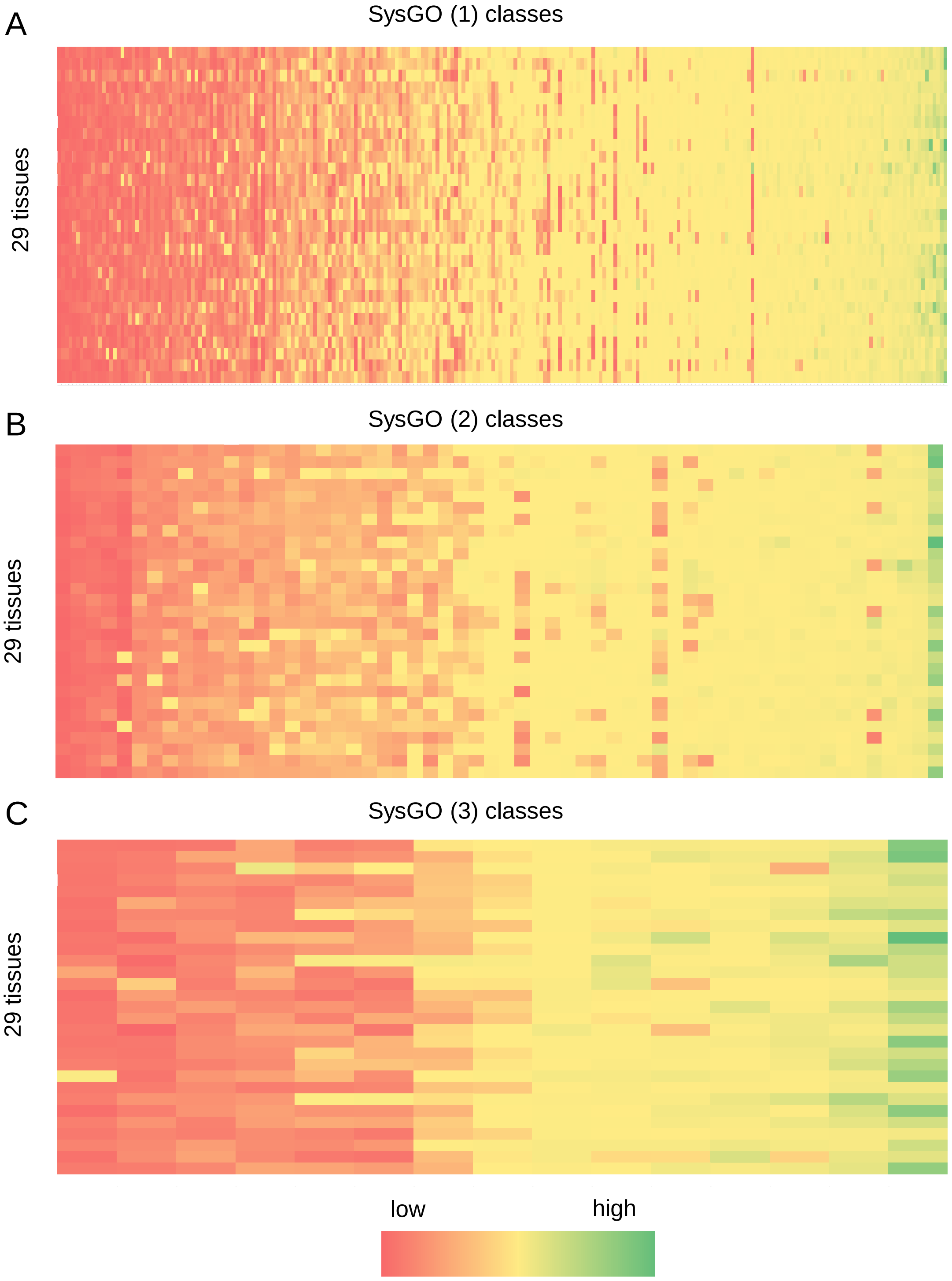


**Figure S6. Protein abundance averages across different functional SysGO classes in 29 human tissues. Related to Figure 3.**

Heat map of protein expression average across functional SysGO classes using a deep-coverage published protein expression dataset (Wang et al., 2019). Expression averages are colored from red (low expression) to green (high expression). Each row corresponds to expression levels in one of the 29 human tissues. The columns correspond to (A) 240 SysGO (level 1) classes, (B) 58 SysGO (level 2) classes, and (C) 15 SysGO (level 3) classes.

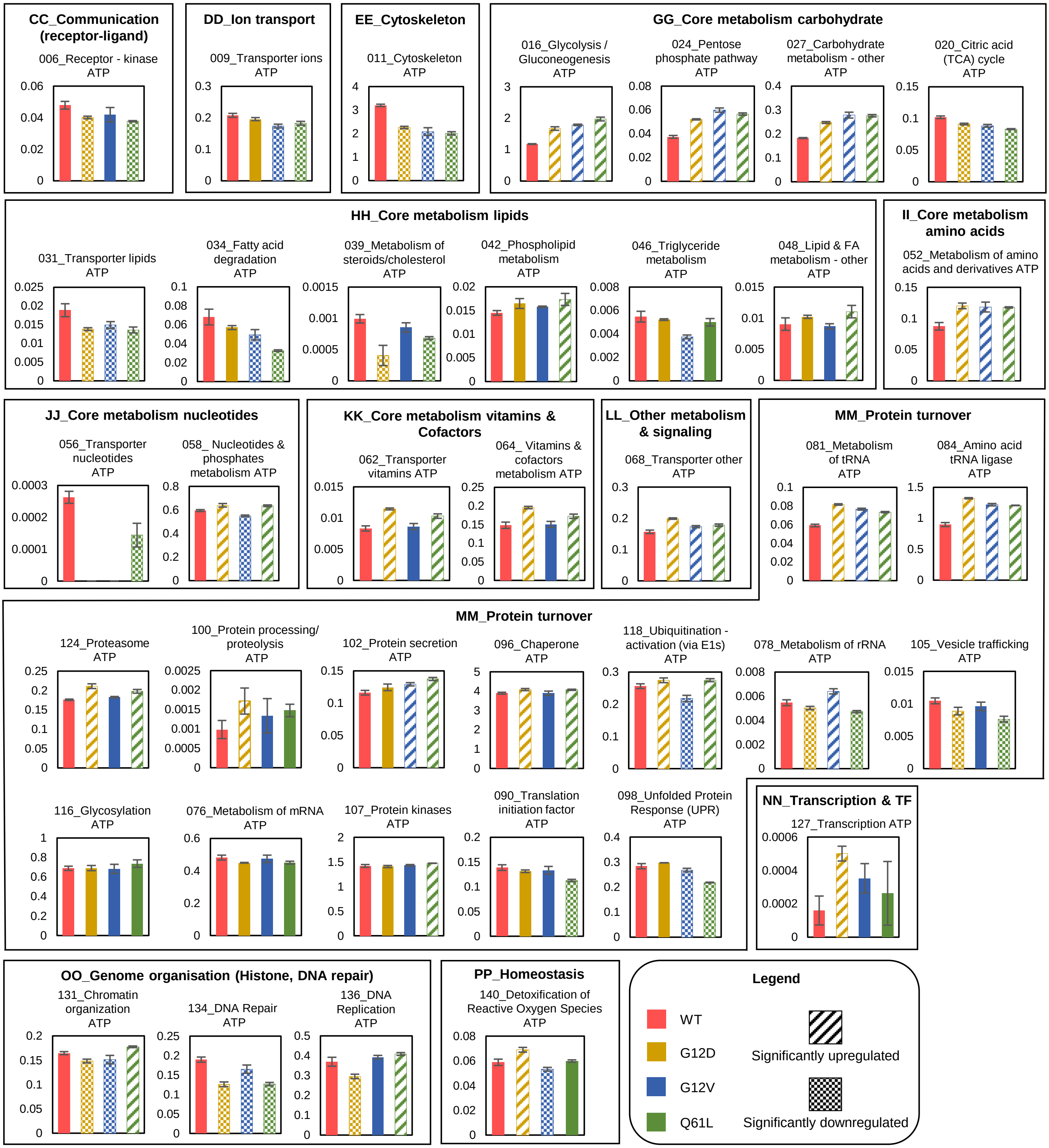


**Figure S7. Protein expression sums of different ATP-requiring enzyme classes based on the EnerSysGO database for MEF WT and mutant cell lines. Related to Figure 3.**

Values are mean ± SD for protein expression data (% of total protein) of three biological replicates where missing values were filled individually in each dataset using the ComPleteROT pipeline. Sum of protein expression was analysed usimg one-way ANOVA followed by Dunnett's *post-hoc* tests. Significant difference ($p < 0.05$) for mutants versus WT are indicated with bar diagrams containing a specific filling pattern (see legend).

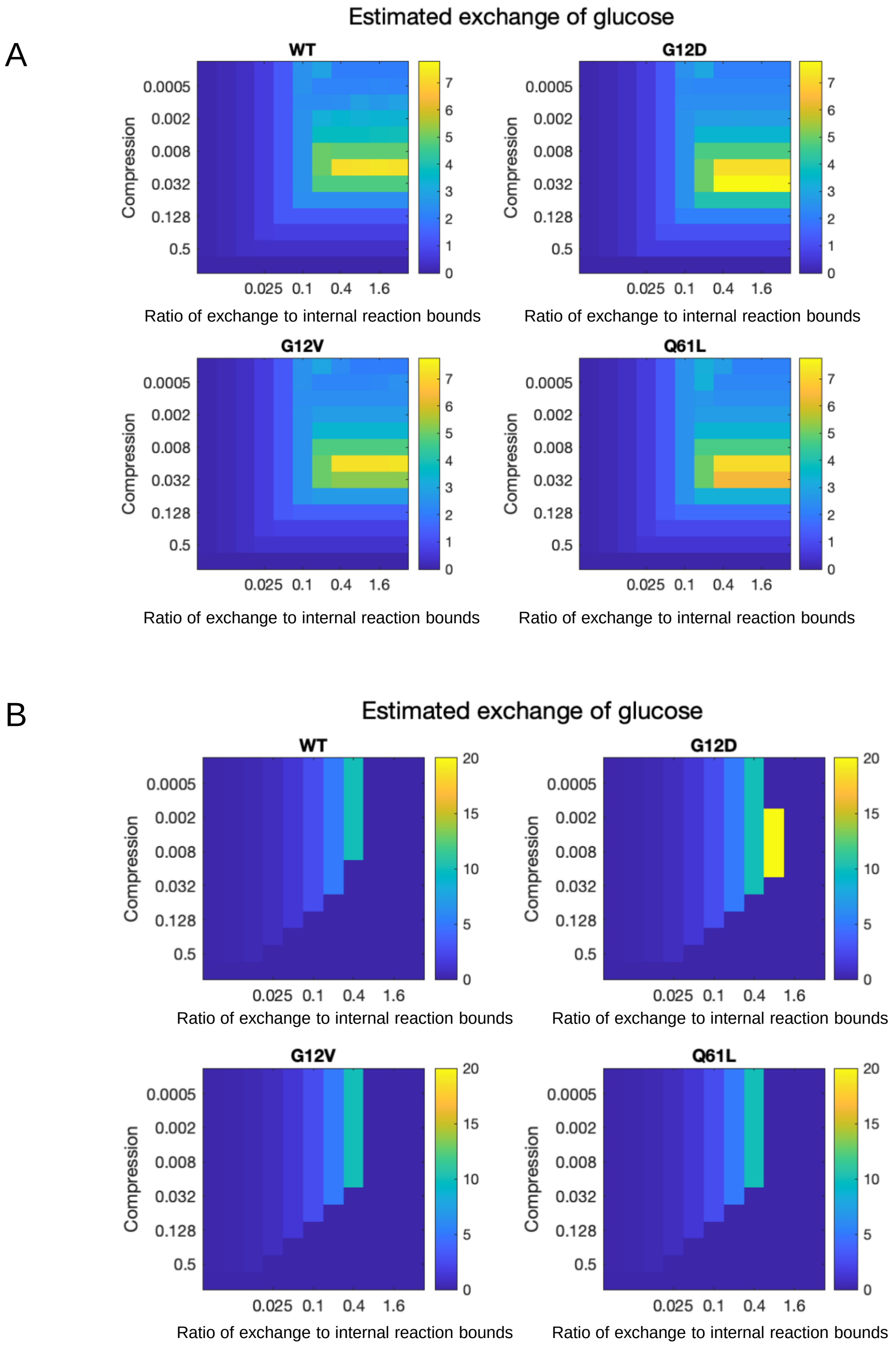


**Figure S8. Results of FBA analysis for exchange of glucose of metabolic models in the four MEF cell lines. Related to Figure 4.**

(A) Results of simulations that use exchange reaction bounds estimated in terms of relative abundances within DMEM for WT, G12D, G12C, and Q61L. The units are arbitrary (fluxes).

(B) Results of simulations that use measured values for glucose and glutamine uptake and lactate and glutamate efflux as exchange reaction bounds but scaled in the same way as for the DMEM plots, that is availability of metabolites in the culture medium relative to enzyme and transporter abundances increases from left to right, for WT, G12D, G12C, and Q61L. Dark areas on the right and bottom right-hand corner represent areas where the FBA could not find a solution. The units are arbitrary (fluxes).

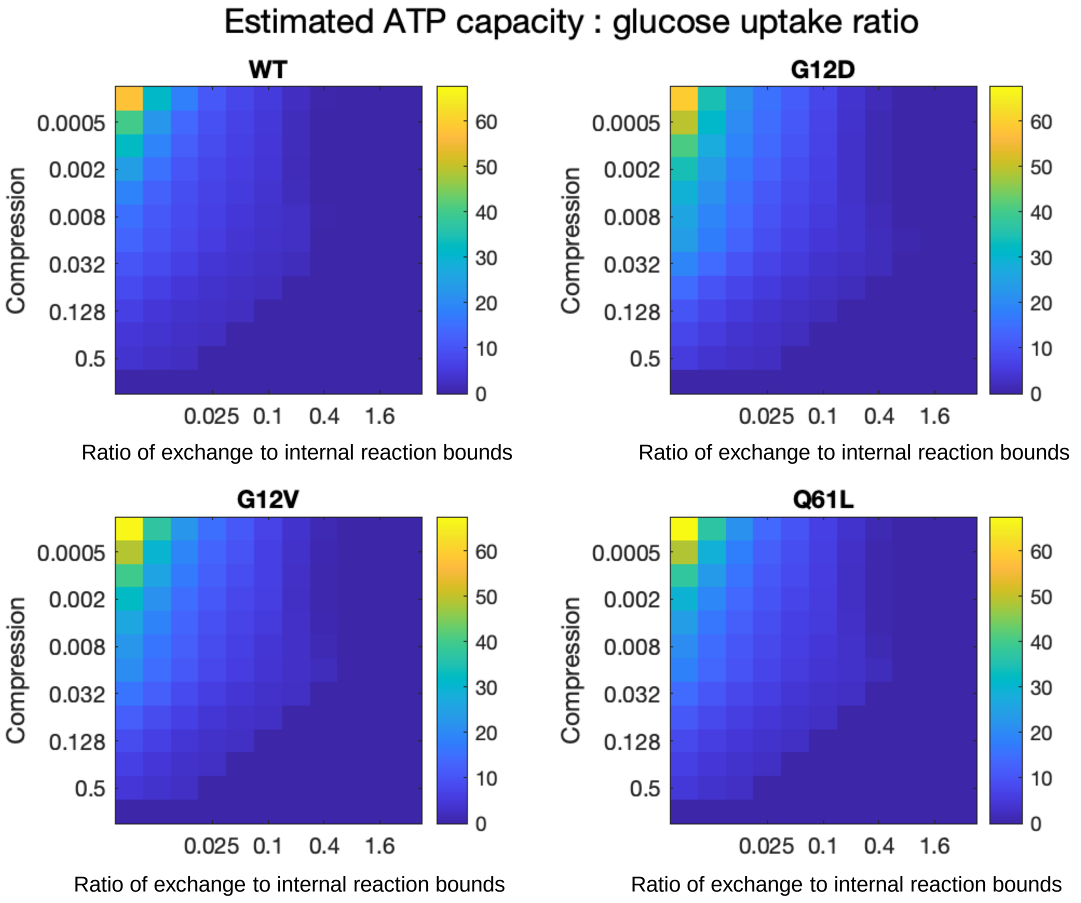


**Figure S9. Results of FBA analysis for ratio of ATP generated to glucose uptake of metabolic models in the four MEF cell lines. Related to Figure 5.**

Results of simulations that use measured values for glucose and glutamine uptake and lactate and glutamate efflux as exchange reaction bounds but scaled in the same way as for the DMEM plots, that is availability of metabolites in the culture medium relative to enzyme and transporter abundances increases from left to right, for WT, 12D, G12C, and Q61L. The peak 'efficiency' is seen in the top left-hand corner where glucose availability is low in comparison to internal fluxes and low-level abundances' impact on the solution are minimized. The units are dimensionless ratios.
